# An integrated design concept evaluation model based on interval valued picture fuzzy set and improved GRP method

**DOI:** 10.1038/s41598-024-57960-9

**Published:** 2024-04-10

**Authors:** Qing Ma, Zhe Chen, Yuhang Tan, Jianing Wei

**Affiliations:** Shandong Jiaotong University, Jinan, 250357 China

**Keywords:** Design concept evaluation, Kano model, Interval-valued picture fuzzy set, Multiplicative AHP method, Entropy of IVPFS, Improved GRP method, Information technology, Computational science, Computational methods

## Abstract

The objective of this research is to enhance the precision and efficiency of design concept assessments during the initial stages of new product creation. Design concept evaluation, which occurs at the end of the conceptual design phase, is a critical step in product development. The outcome of this evaluation significantly impacts the product's eventual success, as flawed design concepts are difficult to remedy in later stages. However, the evaluation of new product concepts is a procedure that encompasses elements of subjectivity and ambiguity. In order to deal with the problem, a novel decision-making method for choosing more logical new product concepts is introduced. Basically, the evaluation process is outlined in three main phases: the construction of evaluation index system for design concept alternatives, the calculation of weights for evaluation criteria and decision-makers, the selection of the best design concept alternatives. These stages are composed of a hybrid method based on kano model, multiplicative analytic hierarchy process (AHP) method, the entropy of IVPFS and improved grey relational projection (GRP) under interval-valued picture fuzzy set (IVPFS). The novel approach integrates the strength of interval-valued picture fuzzy number in handling vagueness, the advantage of multiplicative AHP and the merit of improved GRP method in modelling multi-criteria decision-making. In final, the effectiveness of the proposed model is validated through comparisons with other models. The potential applications of this study include but are not limited to product development, industrial design, and innovation management, providing decision-makers with a more accurate and comprehensive design concept evaluation tool.

## Introduction

New Product Development (NPD) is crucial for manufacturers to excel in competitive markets. As a key corporate function, NPD involves critical decision-making, with design concept evaluation being a standout step. This process assesses potential designs against criteria to select the most viable option. Since a large portion of a product's cost and quality is set in the conceptual phase, accurate evaluations are vital to avoid costly redesigns^1,2^. Effective evaluations also help managers quickly focus on promising ideas, streamlining development and boosting NPD success rates.

In the evaluation process of NPD, the uncertainty and ambiguity arise from the different cognitive levels and experiences of DMs. These factors can generate a negative impact on the evaluation process and the results of design concept. Therefore, how to eliminate information ambiguity is an important issue in product concept design evaluation^3^.

In order to solve the ambiguity and uncertainty of evaluation information for DMs, previous researchers have proposed interval set^4^, rough set^5^ and fuzzy set (FS)^6^ theories. The interval number provides DMs with a clearer understanding of the meaning of design choices. At the same time, it is more helpful for DMs to make wise decisions, considering uncertainty and change. However, interval theory oversimplifies practical problems when dealing with uncertainty, ignoring the fuzziness and probability distribution of parameters. FS, along with its extended forms such as intuitionistic fuzzy sets (IFS)^7^, hesitant fuzzy sets (HFS)^8^, neutrosophic set (NS)^9,10^, pythagorean fuzzy sets^11^, and picture fuzzy sets (PFS)^12^, can compensate for the deficiencies of interval sets. The combination of interval theory and FS can express the degree of uncertainty of parameters within intervals using fuzzy membership functions. Compared to extended forms, FS still falls short in describing the ambiguity and uncertainty of DMs’ evaluation information. For instance, FS only considers membership degrees without taking into account non-membership degrees, hesitation degrees, or degrees of abstention. This may be insufficient to fully describe the DMs’ preferences in practical situations, leading to inaccurate evaluation results.

In order to overcome the above issues, this study proposes a novel and reasonable framework to select design concept schemes. The main innovations and contributions of this study are organized as:

1. The first study applied to the mapping relation between CRs and the evaluation index to determine criteria of design concept.
2. This study effectively proposed the transformation of linguistic values to IVPFN to express DM evaluation information, which solves the uncertainty in the design concept evaluation process.
3. This study proposed improved GRP method to determine the best alternative in product design concept evaluation process.

The subsequent sections of this study are organized as follows: In Section “Literature review”, an overview of the relevant literature is presented. Section “Basic preliminaries” sets out various essential concepts within the IVPFS, introduces fundamental operating principles of IVPFN. Section “Proposed methodology” elaborates a distinctive framework for assessing and selecting design concept alternatives, incorporating the Kano model and an enhanced GRP method with IVPFS. To showcase the applicability of the proposed approach, a case study is expounded upon in Section “Case study”. Section “Conclusion” summarizes the findings of the study and explores potential future applications.

## Literature review

Our research aims to assess design concept alternatives using the Kano model, IVPFS, and an improved GRP method. Consequently, the literature review is divided into three sections: (1) research on the Kano model, (2) research on uncertainty and fuzzy modeling in evaluation information. (3) research on ranking the schemes through improved GRP method under IVPFS.

### Kano model

**Figure 1:**
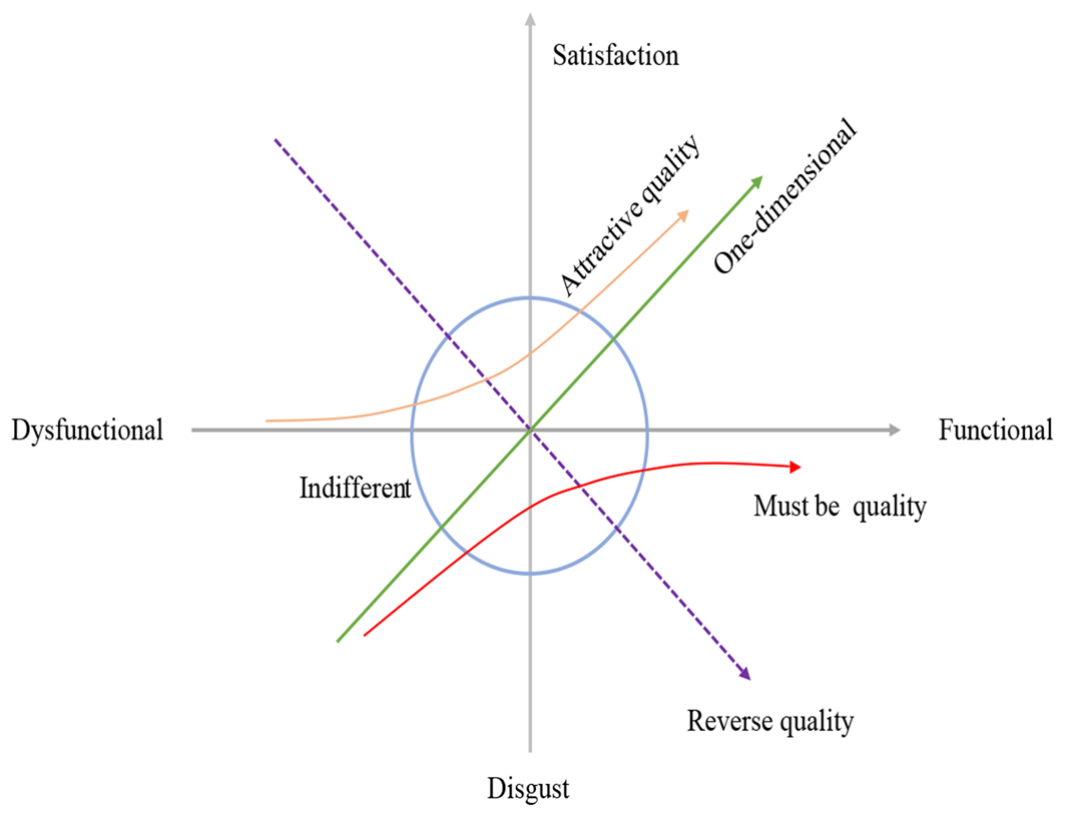
Kano model.

Kano and his colleagues first put forth the Kano model^13^. The Kano model aims to categorize the features of a product or service based on their ability to meet customer needs. In practical terms, the properties of the Kano model can be classified into five groups, as illustrated in Fig. 1 and Table 1.

**Table 1:** Five types of requirements.

Numbers	Types	Specific meanings
1	Must-be attributes (M)	Vital for satisfaction, their absence reduces it, but improving performance minimally impacts overall user contentment
2	One-dimensional attributes (O)	Enhanced functionality boosts it; reduced functionality lowers satisfaction levels
3	Attractive attributes (A)	The lack of these requirements won't lead to customer dissatisfaction. Yet, their presence can markedly enhance customer satisfaction
4	Indifferent attributes (I)	The lack of these requirements won't lead to customer dissatisfaction. Yet, their presence can markedly enhance customer satisfaction
5	Reverse attributes (R)	The presence of these requirements leads to customer dissatisfaction

Applying the Kano model to define quality categories aids designers in understanding customers’ actual requirements. This, in turn, enables more precise control over quality and satisfaction during the product design and development process^14^. Wu et al.^15^ proposed that an evaluation procedure based on the Kano model is mainly to help identify attractive customer requirements (CRs) through the use of the Kano model. To capture CRs and provide inspiring insights for emotional design from the perspective of businesses, Jin et al.^16^ created the Kansei-integrated Kano model. In our research, we utilize the Kano model to categorize CRs, identify the ultimate CRs, and establish the evaluation index system by mapping the connection between CRs and attributes.

### Uncertainty and fuzzy modeling in evaluation information

In the process of design concept evaluation, the fuzziness of individual experience and knowledge of DMs leads to uncertainty in evaluation information^17^. To ensure the accuracy of evaluation results, interval theory and various FS have been introduced, including IFS, NS, Pythagorean fuzzy sets and PFS.

Interval theory represent fuzziness by defining upper and lower bounds. This method can more intuitively describe the uncertainty of DMs regarding evaluation information, especially suitable for situations where precise values are difficult to define. Jiang et al.^18^ proposed a new interval comparison relation and applied it to interval number programming, and established two transformation models for linear and nonlinear interval number programming problems to solve practical engineering problems. Yao et al.^19^ defined an interval number ordering method and its application considering symmetry axis compensation. The feasibility and validity of the method are also verified through examples. However, interval theory also faces the problem of insufficient accuracy, as they typically represent uncertainty through ranges and fail to provide detailed fuzzy membership functions. FS use membership functions to model fuzziness, but their simplification of varying degrees of fuzziness limit their expressive power when dealing with complex design information. IFS emphasize the subjective cognition and experience of DMs. Wang et al.^20^ combined intuitionistic fuzzy sets with the VIKOR method for the project investment decision-making process. Zeng et al.^21^ proposed the weighted intuitionistic fuzzy IOWA weighted average operator. And using the proposed operator, they also developed a procedure for solving multi-attribute group decision-making problems. Nevertheless, they have certain shortcomings, such as the inability to accurately express the attitudes or opinions of DMs including affirmation, neutrality, negation, and rejection. NS theory has more extensive applications than FS and IFS theory. However, the function values of the three membership functions in the NS are subsets of non-standard unit intervals, making it difficult to apply to practical problems. Compared to others, PFS as a novel form of FS, introduces concepts such as membership degree, non-membership degree, neutrality degree, and abstention degree, which more comprehensively considers the psychological state of DMs in evaluation. Membership degree describes the degree of belonging between elements and FS, non membership degree reflects the degree to which elements do not belong to FS, and abstention degree expresses the degree of uncertainty that DMs have about certain elements. This comprehensive consideration of different aspects of information makes the PFS more adaptable and can more accurately and comprehensively reflect the psychological state of DMs in actual decision-making situations, providing more accurate information support for design concept evaluation. Kahraman^22^ proposed proportion-based models for PFS, facilitating the utilization of PFS by incorporating accurate data that more effectively reflects the judgments of DMs. Luo et al.^23^ introduced a novel distance metric for PFS, employing three-dimensional divergence aggregation. This proposed distance metric is then utilized to address MCDM problems. Wang et al.^24^ devised a multi-attributive border approximation area comparison method based on prospect theory in a picture fuzzy environment. The algorithm's applicability is demonstrated through a numerical example, highlighting its advantages.

However, in MCDM, due to the limitations of DMs' understanding of the decision object and the ambiguity of the decision environment, DMs are often faced with situations that are difficult to define precisely, and thus prefer to give an interval number. In order to better deal with this challenge, the IVPFS has been proposed^12^. The innovation of IVPFS lies in its ability to represent membership degree, non-membership degree, neutrality degree, and abstention degree in the form of interval numbers^25,26^. In contrast, the interval-valued Pythagorean fuzzy set is composed of three parts: membership degree, non-membership degree, and hesitancy degree^27,28^. IVPFS can better describe and express the uncertainty and fuzziness of DMs in practical decision-making. This theory is proposed to improve the credibility of decision-making outcomes thus enhancing the usefulness and adaptability of DMs participation in MCDM problems. Cao et al.^29^ proposed an innovative similarity measure for IVPFS, taking into account the impact of the margin of the degree of refusal membership. Mahmood et al.^30^ introduced the interval-valued picture fuzzy frank averaging operator, and discussed their properties. The relationship between IVPFS and other sets is shown in Table 2.

**Table 2:** the relationship between IVPFS and other sets.

Theory	Expression	Literature
Interval set	$\left\{x|a\le x\le b,x\in {\mathbb{R}}\right\}$	^31^
FS	$A=\left\{\left(x,{\mu }_{A}\left(x\right)\right)|x\in X\right\}$	^5^
IFS	$B=\left\{\left(x,{\mu }_{B}\left(x\right),{\eta }_{B}\left(x\right)\right)|x\in X\right\}$	^32^
IVIFS	$C=\left\{\left(x,\left[{\mu }_{C}^{L}\left(x\right),{\mu }_{C}^{U}\left(x\right) \right],\left[{\eta }_{C}^{L}\left(x\right),{\eta }_{C}^{U}\left(x\right) \right]\right)|x\in X\right\}$	^33^
PFS	$D=\left\{\left(x,{\mu }_{D}\left(x\right),{\eta }_{D}\left(x\right),{\nu }_{D}\left(x\right)\right)|x\in X\right\}$	^34^
IVPFS	$E=\left\{\left(x,\left[{\mu }_{E}^{L}\left(x\right),{\mu }_{E}^{U}\left(x\right) \right],\left[{\eta }_{E}^{L}\left(x\right),{\eta }_{E}^{U}\left(x\right) \right],\left[{\nu }_{E}^{L}\left(x\right),{\nu }_{E}^{U}\left(x\right) \right]\right)|x\in X\right\}$	^35^

### Improved grey relational projection method

In the process of evaluating design concepts, one must choose a favorite from a multitude of options, a task that constitutes a MCDM issue. Traditional methods for solving the MCDM problem, including the AHP, TOPSIS method, EDAS method, and VIKOR method, which have the unique advantage of targeting specific decision scenarios. However, these methods generally have limitations in dealing with the early stages of design concept. As a multi-factor statistical analysis method, the GRP method excels in dealing with correlations between attributes. The main reasons for applying the GRP method to design concept evaluation are as follows. The GRP method's key benefits include easy-to-understand calculations, high accuracy, and reliance on actual data. In the decision-making process of design concept evaluation, each attribute is not independent of the others. Although the internal relationship is not clear, there is actually some correlation. In essence, it is a grey relationship. Therefore, in decision analysis of such a system, it is actually a grey MCDM problem. Decision making in the GRP approach is a mapping of the set of decision metrics. Once the set of attributes is identified, alternatives can be identified. This approach combines the effects of the entire decision indicator space. Especially when the attributes have discrete sample data, the GRP method avoids unilateral bias, i.e., the bias that arises from comparing a single attribute for each alternative, and thus integrates the analysis of the relationships between the indicators, reflecting the impact of the entire indicator space. Since most GRP methods are based on a single base point (the ideal alternative), our study builds on the existing literature and improves on the GRP method by determining the final score for each design alternative based on the IVPFS.

Table 3 contains a summary that compares the proposed technique to other multi-criteria concept evaluation approaches. These scholars investigated a number of potential aspects that could influence the decision-making process. However, significant obstacles remain in concept evaluation, which is the focus of this paper's research. To address the above issues thoroughly, a design concept evaluation technique is provided that incorporates the kano model, mapping relation, IVPFS, and improved GRP method to produce the best concept.

**Table 3:** literature review table.

Literature	Mapping relation	Uncertainty	Attributes Correlation	Information aggregation	Case study
Akay et al.^36^		**√**		**√**	Adhesive tape dispenser
Zhu et al.^37^		**√**		**√**	Lithography tool
Shidpour et al.^5^		**√**	**√**		Mobile product
Aikhuele et al.^38^		**√**			New product
Tiwari et al.^39^		**√**		**√**	New product
Hayat et al.^40^		**√**			New product
Song et al.^41^		**√**	**√**		Intelligent products
Qi et al.^42^		**√**			Customer-oriented product
Zhou et al.^43^		**√**			smart product service system
Huang et al.^44^		**√**		**√**	Refrigerator design
Yang et al.^45^		**√**			Product-service system
Proposed method	**√**	**√**	**√**	**√**	Yacht design

## Basic preliminaries

We review several fundamental ideas in this section to provide some required background knowledge.

### Construct the index of design concept evaluation

The Kano model finds extensive application in the realm of MCDM. The creation of the design concept evaluation indicator system, as proposed in this paper, primarily involves the following steps. First, relevant CRs for evaluating the design concept scheme are gathered. Then, employing the Kano model, requirement attributes are assessed, filtering out less critical requirements and retaining the most important ones. Ultimately, the evaluation index system for the design concept is formulated by establishing the mapping relationship between requirements and the evaluation indices.

Initially, we gathered and organized the primary CRs for the design concept schemes, as illustrated in Table 4.

**Table 4:** Collection of the initial CRs of the design concept schemes.

CR1	CR4	$\cdots$	CR7
CR2	CR5	$\cdots$	CR8
CR3	CR6	$\cdots$	CRn

Next, we designed a questionnaire for CRs considering both a product with and without the same functional requirement. Each question in the questionnaire includes a description of the functional requirement to aid customers in comprehending its significance. To ensure uniform understanding among users, we provided consistent explanations for the meaning of the options in the questionnaire. This facilitates easy comprehension for users, allowing them to indicate their responses effectively. The design of the Kano questionnaire is presented in Table 5.

**Table 5:** Kano questionnaire.

Product/Service	Like it	Must-be	Neutral	Live-with	Dislike
Functional		√			
Dysfunctional					√

Subsequently, we processed the feedback data from the returned questionnaires. Quantifying the two dimensions, namely “with function” and “without function,” we obtained an overlapping result by referencing Table 6 for the options corresponding to the scores. This approach allows us to discern the type of CRs.

**Table 6:** Kano evaluation table.

Functional	Dysfunctional				
	Like it	Must-be	Neutral	Live-with	Dislike
Like it	Q	A	A	A	O
Must-be	R	I	I	I	M
Neutral	R	I	I	I	M
Live-with	R	I	I	I	M
Dislike	R	R	R	R	Q

*Q* questionable, *A* attractive, *O* one-dimensional, *M* must-be, *I* indifference, *R* reverse.

**Figure 2:**
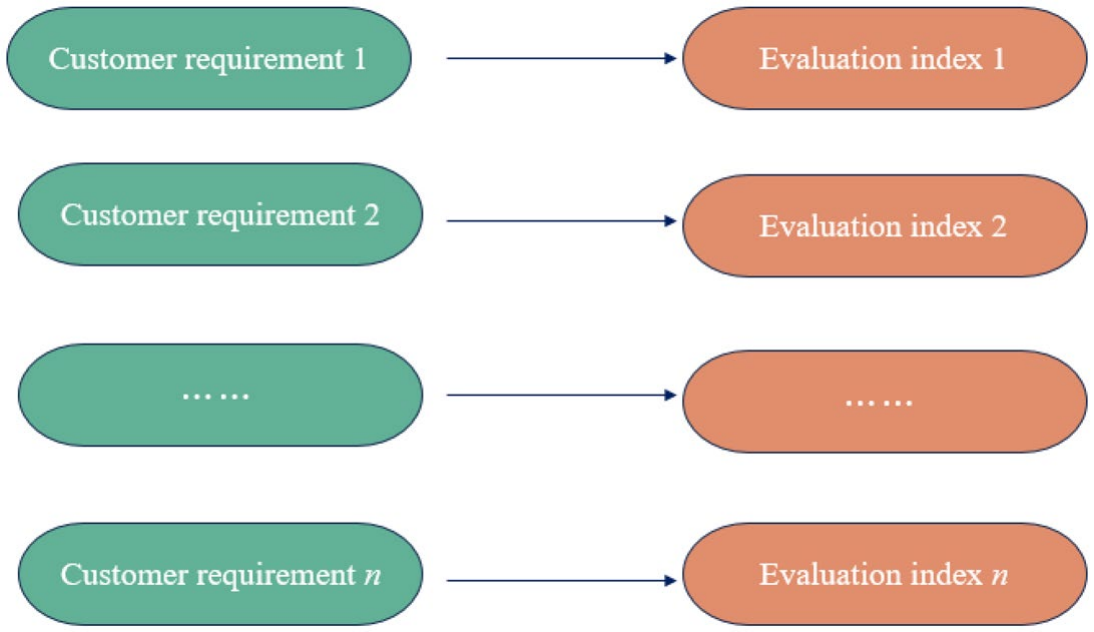
The mapping relation between CRs and the evaluation index.

The CRs established in this study are derived from an analysis of issues identified by research customers during product use in specific scenarios. The fulfillment of these requirements indicates customer satisfaction with the product’s usage. Consequently, the CRs serve as indicator factors for users to assess the design concept. The mapping relationship between the two is depicted in Fig. 2.

Ultimately, by excluding indicators that fall outside the scope of CRs, the evaluation index system for design concept alternatives based on CRs can be established.

### The multiplicative AHP method

AHP is widely used for attribute weight determination, relying on an additive value function and making decisions through pairwise comparisons. However, AHP may encounter rank reversals, potentially leading to incorrect results. An enhanced method, the multiplicative AHP, addresses this by introducing a structured hierarchical approach, mitigating rank reversal issues associated with the original AHP^46^. In the multiplicative AHP method, DMs are tasked with comparing schemes in pairs and rendering decisions based on attributes. Subsequently, these judgments are aggregated, and the criteria weights are calculated using the compiled information^47^. The specific steps of the multiplicative AHP approach are as follows: Assume there are $t$ experts in the decision-making group $E$, denoted as $E=\{{e}_{1},{e}_{2},\dots ,{e}_{t}\}$. ${A}_{j}$ and ${A}_{k}$ are two alternatives, the expert’s preference of ${A}_{j}$ and ${A}_{k}$ are present to two stimuli ${S}_{j}$ and ${S}_{k}$, and expert $e$ in group $E$ is assigned to make pairwise comparisons according to an attribute by the linguistic information in Table 7. The linguistic information is then converted into numerical scales denoted as ${\delta }_{jke}$. Comparisons made by expert $e$ are denoted as ${\delta }_{12e}$,${\delta }_{13e}$,…,${\delta }_{23e}$, ${\delta }_{24e}$, … , ${\delta }_{(t-1)(t)e}$. To eliminate the bias caused by the individual emotional factor, the comparisons with the expert themself are invalid and not included in the evaluation. Hence, for expert group $E$, the maximum number of valid judgements is $(t-1)(t-2)/2$.

**Table 7:** Linguistic information and corresponding numerical scale in pairwise comparison.

Compared with ${S}_{j}$, ${S}_{k}$ is	Extremely poor	Very poor	Poor	Medium poor	Indifferent	Medium good	Good	Very good	Extremely good
Numerical scale (${\delta }_{jke}$)	− 8	− 6	− 4	− 2	0	2	4	6	8

*Step 1*: From the judgements made by the experts in group $E$, establish the decision matrix ${\{r}_{jke}\}$ by combining the judgements of the experts, denoted as:

1 $$\begin{array}{c}{r}_{jke}=\mathit{exp}\left(\gamma {\delta }_{jke}\right) \end{array}$$

Here the variant $\mathrm{\gamma d}$ enotes a scale parameter commonly equal to ${\text{ln}}2$, $j=\mathrm{1,2},\dots ,t$.

*Step 2*: Determine the approximate vector $p$ of stimulus values by the logarithmic least-squares method:

2 $$\begin{array}{c}\sum\limits_{j<k}\sum_{e\in {S}_{jk}}{\left(\mathit{ln}{r}_{jke}-\mathit{ln}{p}_{j}+\mathit{ln}{p}_{k}\right)}^{2} \end{array}$$

where ${S}_{jk}$ denotes the expert set who judged ${S}_{j}$ with respect to ${S}_{k}$. Let ${\lambda }_{j}={\text{ln}}{p}_{j}$,${\lambda }_{k}={\text{ln}}{p}_{k}$ and ${q}_{jke}={\text{ln}}{r}_{jke}=\upgamma {\delta }_{jke}$. Rewrite Eq. (2) with these substitutions as

3 $$\begin{array}{c}\sum\limits_{j<k}{{\sum }_{e\in {S}_{jk}}\left({q}_{jke}-{\lambda }_{j}+{\lambda }_{k}\right)}^{2} \end{array}$$

Let ${N}_{jk}$ be the cardinality of the expert set ${S}_{jk}$, Eq. (3) can be transferred to

4 $$\begin{array}{*{20}l} {\lambda _{j} \mathop \sum \limits_{{k = 1,k \ne j}}^{t} N_{{jk}} - \lambda _{k} \mathop \sum \limits_{{k = 1,k \ne j}}^{t} N_{{jk}} = \mathop \sum \limits_{{k = 1,k \ne j}}^{t} \mathop \sum \limits_{{e \in S_{{jk}} }} q_{{jke}} } \\ \end{array}$$

If the comparisons including the expert are not considered, then

5 $$\begin{array}{c}{N}_{jk}=t-2 \end{array}$$

As the maximum pairwise comparison is $\left(t-1\right)\left(t-2\right)$, Eq. (4) can be rewritten as

6 $$\begin{array}{*{20}l} {\lambda _{j} \left( {t - 1} \right)\left( {t - 2} \right) - \mathop \sum \limits_{{k = 1,k \ne j}}^{t} \left( {t - 2} \right)\lambda _{k} = \mathop \sum \limits_{{k = 1,k \ne j}}^{t} \mathop \sum \limits_{{e \in S_{{jk}} }} q_{{jke}} } \\ \end{array}$$

A simplified style of the equation is

7 $$\begin{array}{*{20}c} {W_{j} t\left( {t - 2} \right) - \left( {t - 2} \right)\mathop \sum \limits_{{k = 1,k \ne j}}^{t} w_{k} = \mathop \sum \limits_{{k = 1,k \ne j}}^{t} \mathop \sum \limits_{{e = 1,e \ne j}}^{t} q_{{jke}} } \\ \end{array}$$

*Step 3*: From Table 7, for ${A}_{k}$ and ${A}_{j}$, the sum of the numerical scale ${\delta }_{jke}$ and ${\delta }_{kje}$ is equal to 0, which means ${q}_{jky}=-{q}_{kjy}$. Hence ${q}_{jjy}=0$, so let ${\sum }_{k=1,k\ne j}^{t}{{\text{w}}}_{k}=0$. Equation (7) can be further simplified and ${\lambda }_{j}$ can be determined as

8 $$\lambda _{j} = [t\left( {t - 2} \right)]^{{ - 1}} ~\mathop \sum \limits_{{k = 1,k \ne j}}^{t} \mathop \sum \limits_{{e = 1,e \ne j}}^{t} q_{{jke}}$$

Hence, the ${p}_{j}$ can be computed as:

9 $$\begin{array}{c}{p}_{j}=\mathit{exp}\left({\lambda }_{j}\right)\end{array}$$

*Step 4*: Calculate the normalized weight ${w}_{j}$ determined by multiplicative AHP as

10 $$\begin{array}{c}{w}_{j}=\frac{{p}_{j}}{\sum\limits{p}_{j}} \end{array}$$

### Interval-valued picture fuzzy set

In 2013, Cuong et al. proposed a new concept of IVPFN to quantify vague DMs’perception based on the basic principles of IVPFS. IVPFN more accurately captures the genuine insights of DMs, thus increasing the objectivity of the evaluation data. According to Cuong et al., the definition of IVPFS is shown below.

#### Definition 1

^12^ Considering a designated domain of discourse denoted as $X$, where *U* [0,1] signifies the set of subintervals within the interval [0,1], and $x\ne 0$ is a given set. In this study, the IVPFS is defined as follows:

11 $$\begin{array}{c}B=\left\{\langle x,{\varrho }_{B}\left(x\right),{\xi }_{B}\left(x\right),{\upsilon }_{B}\left(x\right)\rangle \mid x\in X\right\} \end{array}$$

The intervals ${\varrho }_{B}\left(x\right),{\xi }_{B}\left(x\right),{\upsilon }_{B}\left(x\right)$ represent positive, negative and neutral membership degrees of $B$, Additionally, ${\varrho }_{B}^{L}\left(x\right), {\varrho }_{B}^{U}\left(x\right), {\xi }_{B}^{L}\left(x\right), {\xi }_{B}^{U}\left(x\right), {\upsilon }_{B}^{L}\left(x\right), {\upsilon }_{B}^{U}\left(x\right)$ represent the lower and upper end points. Consequently, the IVPFS *B* can be expressed as:

12 $$\begin{array}{c}B=\left\{\langle x,\left[{\varrho }_{B}^{L}\left(x\right), {\varrho }_{B}^{U}\left(x\right)\right],\left[{\xi }_{B}^{L}\left(x\right), {\xi }_{B}^{U}\left(x\right)\right],\left[{\upsilon }_{B}^{L}\left(x\right), {\upsilon }_{B}^{U}\left(x\right)\right]\rangle \mid x\in X\right\} \end{array}$$

where ${\varrho }_{B}^{L}\left(x\right)\ge 0, {\xi }_{B}^{L}\left(x\right)\ge 0 \& {\upsilon }_{B}^{L}\left(x\right)\ge 0$ and $0\le {\varrho }_{B}^{U}\left(x\right)+{\xi }_{B}^{U}\left(x\right)+{\upsilon }_{B}^{U}\left(x\right)\le 1$.Refusal membership degree expressed by ${\sigma }_{B}$ can be calculated using the Eq. (13).

13 $$\begin{array}{c}{\sigma }_{B}=\left[{\sigma }_{B}^{L}\left(x\right), {\sigma }_{B}^{U}\left(x\right)\right]=\left[1-\left({\varrho }_{B}^{U}\left(x\right)+{\xi }_{B}^{U}\left(x\right)+{\upsilon }_{B}^{U}\left(x\right)\right),1-\left({\varrho }_{B}^{L}\left(x\right)+{\xi }_{B}^{L}\left(x\right)+{\upsilon }_{B}^{L}\left(x\right)\right) \right] \end{array}$$

#### Definition 2

^48^ Let that ${{\text{B}}}_{{\text{i}}}=(\left[{\varrho }_{{\text{i}}}^{{\text{L}}},{\varrho }_{{\text{i}}}^{{\text{U}}}\right],\left[{\xi }_{{\text{i}}}^{{\text{L}}},{\xi }_{{\text{i}}}^{{\text{U}}}\right],\left[{\upsilon }_{{\text{i}}}^{{\text{L}}},{\upsilon }_{{\text{i}}}^{{\text{U}}}\right])({\text{i}}=\mathrm{1,2},\ldots ,{\text{n}})$ be the IVPFN, $\Omega$ is the set of IVPFNs. $\upomega ={\left({\upomega }_{1},{\upomega }_{2},\ldots ,{\upomega }_{{\text{n}}}\right)}^{{\text{T}}}$ as the weight vector of them, a mapping IVPFOWIA: ${\Omega }^{{\text{n}}}\to\Omega$ of dimension *n* is an IVPFOWIA operator, with $\sum_{i=1}^{n}{\omega }_{i}=1$, ${\omega }_{i}=\left[\mathrm{0,1}\right]$. Then,

14 $$\begin{array}{*{20}l} {IVPFOWIA\left( {B_{1} ,B_{2} , \ldots ,B_{n} } \right) = \oplus \mathop {\left( {\omega_{i} B_{i} } \right)}\limits_{i = 1}^{n} } \\ \end{array}$$

#### Definition 3

^49^ For two IVPFNs $A={(\varrho }_{A}\left(x\right),{\xi }_{A}\left(x\right),{\upsilon }_{A}\left(x\right))$ and ${B=(\varrho }_{B}\left(x\right),{\xi }_{B}\left(x\right),{\upsilon }_{B}\left(x\right))$. $\lambda$ as a scalar value $\lambda >0$. The following shows the basic and significant operations of IVPFS:

1. $$A\oplus B=\left(\left[{\varrho }_{A}^{L}+{\varrho }_{B}^{L}-{\varrho }_{A}^{L}{\varrho }_{B}^{L},{\varrho }_{A}^{U}+{\varrho }_{B}^{U}-{\varrho }_{A}^{U}{\varrho }_{B}^{U}\right],\left[{\xi }_{A}^{L}{\xi }_{B}^{L},{\xi }_{A}^{U}{\xi }_{B}^{U}\right],\left[{\upsilon }_{A}^{L}{\upsilon }_{B}^{L},{\upsilon }_{A}^{U}{\upsilon }_{B}^{U}\right]\right)$$
2. $$A\otimes B=([{\varrho }_{A}^{L}{\varrho }_{B}^{L},{\varrho }_{A}^{U}{\varrho }_{B}^{U}],[{\xi }_{A}^{L}+{\xi }_{B}^{L}-{\xi }_{A}^{L}{\xi }_{B}^{L},{\xi }_{A}^{U}+{\xi }_{B}^{U}-{\xi }_{A}^{U}{\eta }_{B}^{U}],[{\upsilon }_{A}^{L}+{\upsilon }_{B}^{L}-{\upsilon }_{A}^{L}{\upsilon }_{B}^{L},{\upsilon }_{A}^{U}+{\upsilon }_{B}^{U}-{\upsilon }_{A}^{U}{\upsilon }_{B}^{U}])$$
3. $${A}^{\lambda }=\left(\left[{\left({\varrho }_{A}^{L}\right)}^{\lambda },{\left({\varrho }_{A}^{U}\right)}^{\lambda }\right],\left[1-{\left(1-{\xi }_{A}^{L}\right)}^{\lambda },1-{\left(1-{\xi }_{A}^{U}\right)}^{\lambda }\right],\left[1-{\left(1-{\upsilon }_{A}^{L}\right)}^{\lambda },1-{\left(1-{\upsilon }_{A}^{U}\right)}^{\lambda }\right]\right)$$
4. $$\lambda A=\left(\left[1-{\left(1-{\varrho }_{A}^{L}\right)}^{\lambda },1-{\left(1-{\varrho }_{A}^{U}\right)}^{\lambda }\right],\left[{({\xi }_{A}^{L})}^{\lambda },{({\xi }_{A}^{U})}^{\lambda }\right],\left[{({\upsilon }_{A}^{L})}^{\lambda },{({\upsilon }_{A}^{U})}^{\lambda }\right]\right)$$

#### Definition 4

^30^ Let ${B}_{i}=(\left[{\varrho }_{{\text{i}}}^{{\text{L}}},{\varrho }_{{\text{i}}}^{{\text{U}}}\right],\left[{\xi }_{{\text{i}}}^{{\text{L}}},{\xi }_{{\text{i}}}^{{\text{U}}}\right],\left[{\upsilon }_{{\text{i}}}^{{\text{L}}},{\upsilon }_{{\text{i}}}^{{\text{U}}}\right])$ be an IVPFN, then the score function $SF\left({B}_{i}\right)$ and the accuracy function $AF\left({B}_{i}\right)$ of the IVPFNs can be described as:

15 $$\begin{aligned} SF\left( {B_{i} } \right) & = \frac{{\varrho_{i}^{L} - \xi_{i}^{L} - \upsilon_{i}^{L} + \varrho_{i}^{U} - \xi_{i}^{U} - \upsilon_{i}^{U} }}{3},S\left( {B_{i} } \right) \in \left[ { - 1,1} \right] \\ AF\left( {B_{i} } \right) & = \frac{{\varrho_{i}^{L} + \xi_{i}^{L} + \upsilon_{i}^{L} + \varrho_{i}^{U} + \xi_{i}^{U} + \upsilon_{i}^{U} }}{3},H\left( {B_{i} } \right) \in \left[ {0,1} \right] \\ \end{aligned}$$

Based on the $SF\left({B}_{i}\right)$ and $AF$ of each IVPFN, the comparison rules^50^ between two IVPFNs are given as follows:

For any two IVPFNs ${B}_{1}, {B}_{2}$,

(i) If $$SF\left({B}_{1}\right)> SF\left({B}_{2}\right)$$, then $${B}_{1}>{ B}_{2}$$;
(ii) If $SF\left({B}_{1}\right)= SF\left({B}_{2}\right)$, then
  ① If $$AF\left({B}_{1}\right)> AF\left({B}_{2}\right)$$, then $${B}_{1}>{ B}_{2};$$
  ② If $$AF\left({B}_{1}\right)= AF\left({B}_{2}\right)$$, then $${B}_{1}={ B}_{2}$$.

#### Definition 5

Let ${B}_{1}=\left(\left[{\varrho }_{1}^{{\text{L}}},{\varrho }_{1}^{{\text{U}}}\right], \left[{\xi }_{1}^{{\text{L}}},{\xi }_{1}^{{\text{U}}}\right], \left[{\upsilon }_{1}^{{\text{L}}},{\upsilon }_{1}^{{\text{U}}}\right]\right)$ and ${B}_{2}=(\left[{\varrho }_{2}^{{\text{L}}},{\varrho }_{2}^{{\text{U}}}\right], \left[{\xi }_{2}^{{\text{L}}},{\xi }_{2}^{{\text{U}}}\right],\left[{\upsilon }_{2}^{{\text{L}}},{\upsilon }_{2}^{{\text{U}}}\right])$ represent two IVPFNs, The Hamming distance between ${B}_{1}$ and ${B}_{2}$ is defined as follows:

16 $$\begin{array}{c}{D}_{H}\left({B}_{1},{B}_{2}\right)=\frac{1}{6}\left\{|{\varrho }_{1}^{{\text{L}}}-{\varrho }_{2}^{L}\left|+{|\varrho }_{1}^{U}-{\varrho }_{2}^{U}\right|+{|\xi }_{1}^{L}-{\xi }_{2}^{L}\left|+{|\xi }_{1}^{U}-{\xi }_{2}^{U}\right|+{|\upsilon }_{1}^{L}-{\upsilon }_{2}^{L}\left|+{|\upsilon }_{1}^{U}-{\upsilon }_{2}^{U}\right|\right\} \end{array}$$

The Euclidean distance of ${B}_{1}$ and ${B}_{2}$ is as follows:

17 $$\begin{array}{*{20}c} {D_{E} \left( {B_{1} ,B_{2} } \right) = \frac{1}{6}\sqrt {\left\{ {\left( {|\varrho_{1}^{L} - \varrho_{2}^{L} \left| { + |\varrho_{1}^{U} - \varrho_{2}^{U} } \right|} \right)^{2} + \left( {|\xi_{1}^{L} - \xi_{2}^{L} \left| { + |\xi_{1}^{U} - \xi_{2}^{U} } \right|} \right)^{2} + \left( {|\upsilon_{1}^{L} - \upsilon_{2}^{L} \left| { + |\upsilon_{1}^{U} - \upsilon_{2}^{U} } \right|} \right)^{2} } \right\}} } \\ \end{array}$$

### The entropy of interval-valued picture fuzzy set

In this section, the entropy of IVPFS method is used to calculate criteria weights^48^. This method can handle uncertainty more flexibly and effectively capture measurement errors and fuzziness in practical problems by describing the membership degree of criteria through intervals. The specific calculation formula is as follows:

18 $$\begin{array}{c}{E}_{j}=\frac{1}{m}\sum\limits_{i=1}^{m}\begin{array}{c}\frac{\left(3-\left|{\varrho }_{B}^{L}\left({x}_{i}\right)-{\xi }_{B}^{L}\left({x}_{i}\right)-{\upsilon }_{B}^{L}\left({x}_{i}\right)\right|-\left|{\varrho }_{B}^{U}\left({x}_{i}\right)-{\xi }_{B}^{U}\left({x}_{i}\right)-{\upsilon }_{B}^{U}\left({x}_{i}\right)\right|\right)\left(3+{\sigma }_{B}^{L}\left({x}_{i}\right)+{\sigma }_{B}^{U}\left({x}_{i}\right)\right)}{9}\\ \end{array} \end{array}$$

Finally, use Eq. (19) to calculate the weight of the criteria.

19 $$\begin{array}{c}{w}_{j}=\frac{1-{E}_{j}}{\sum_{j=1}^{m}1-{E}_{j}} \end{array}$$

for all $$j=\mathrm{1,2},\ldots ,n.$$

## Proposed methodology

**Figure 3:**
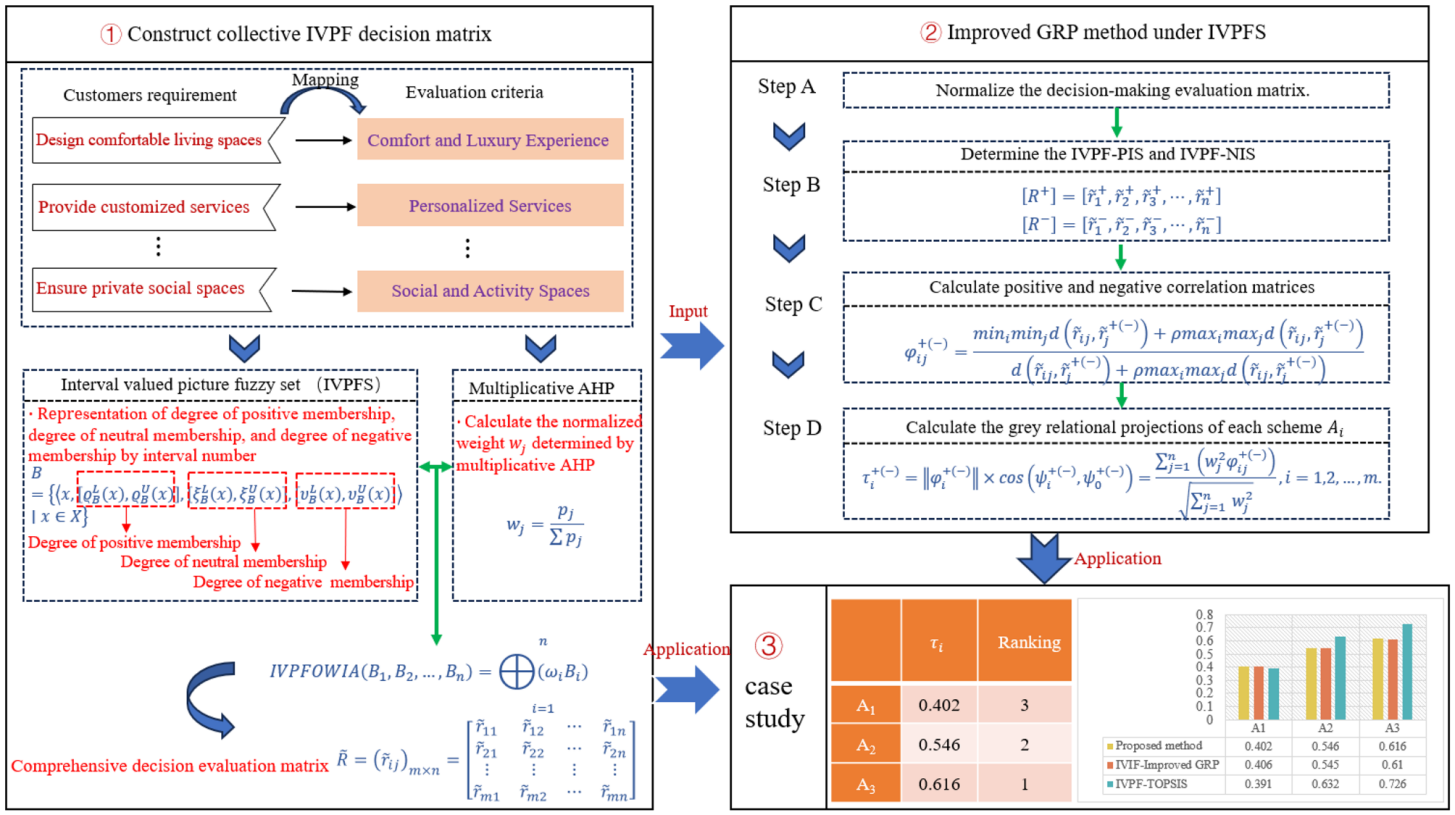
The process of the improved GRP method based on IVPFS.

In this section, we introduce a new framework for selecting yacht design alternatives based on IVPFS and the enhanced GRP technique. The procedural phases of the IVPFS-Improved GRP method are illustrated in Fig. 3, comprising three stages: (1) Construct the collective IVPF decision matrix, (2) Enhance the GRP method under IVPFS theory, and (3) case study. In phase 1, the evaluation index system of the design concept is established using the Kano model, and the weight of each DM is computed through the multiplicative AHP method. With the help of IVPFOWIA, the collective IVPF decision matrix is formulated. In phase 2, the GRP technique is improved within the context of IVPFS to calculate the relative grey relational projection for each alternative. Finally, in phase 3, leveraging the outcomes from phases 1 and 2, the final ranking of different design concept schemes is determined.

For the MCDM problem of design concept evaluation, we denote the set of DMs as $D=\left\{{D}_{1},{D}_{2},\dots ,{D}_{k}\right\}$, the set of design criteria $C=\left\{{C}_{1},{C}_{2},\cdots ,{C}_{n}\right\}$, and the set of design schemes as $A=\left\{{A}_{1},{A}_{2},\dots ,{A}_{m}\right\}$. The weights of design criteria are presented by $w=({w}_{1},{w}_{2},\cdots ,{w}_{j})$, where $\sum_{{\text{j}}=1}^{{\text{n}}}{{\text{w}}}_{{\text{j}}}=1, 0\le {{\text{w}}}_{{\text{j}}}\le 1$. The next sections discuss the specifics of the established design alternative evaluation model based on these assumptions.

### Phase 1: Construct the collective IVPF decision matrix

*Step 1*: Establish the evaluation index evaluation system of design concept by the Kano model.

*Step 2*: Generate the IVPF decision matrix for each DM.

20 $$\begin{array}{c}{R}^{(k)}={\left({r}_{ij}^{(k)}\right)}_{m\times n}=\left[\begin{array}{cccc}{r}_{11}^{(k)}& {r}_{12}^{(k)}& \cdots & {r}_{1n}^{(k)}\\ {r}_{21}^{(k)}& {r}_{22}^{(k)}& \cdots & {r}_{2n}^{(k)}\\ \vdots & \vdots & \vdots & \vdots \\ {r}_{m1}^{(k)}& {r}_{m2}^{(k)}& \cdots & {r}_{mn}^{(k)}\end{array}\right] \end{array}$$

where ${r}_{ij}^{(k)}=\left\{\left[{\varrho }_{ij}^{L(k)}, {\varrho }_{ij}^{U(k)}\right],\left[{\xi }_{ij}^{L(k)}, {\xi }_{ij}^{U(k)}\right],\left[{\upsilon }_{ij}^{L(k)}, {\upsilon }_{ij}^{U(k)}\right]\right\}$ represents an IVPFN. this IVPFN signifies the evaluation value of the alternatives ${A}_{i}$ concerning the criterion ${C}_{j}$ as provided by the DM ${D}_{k}\in D$. And

21 $$\left[{\varrho }_{ij}^{L(k)}, {\varrho }_{ij}^{U(k)}\right]\subseteq \left[\mathrm{0,1}\right], \left[{\xi }_{ij}^{L(k)}, {\xi }_{ij}^{U(k)}\right]\subseteq \left[\mathrm{0,1}\right], \left[{\upsilon }_{ij}^{L(k)}, {\upsilon }_{ij}^{U(k)}\right]\subseteq \left[\mathrm{0,1}\right], {\varrho }_{ij}^{U(k)}+ {\xi }_{ij}^{U(k)}+ {\upsilon }_{ij}^{U(k)}\le 1$$

To specify each ${r}_{ij}^{(k)}$, a 5-scale evaluation was conducted throughout this process. Table 8 illustrates the details of these linguistic scales and their IVPFN equivalents.

**Table 8:** Linguistic scales and interval-valued picture fuzzy numbers for alternatives^51^.

Linguistic Scales	Interval-Valued Picture Fuzzy Numbers
Very High (VH)	$\left(\left[\mathrm{0.75,0.80}\right], \left[\mathrm{0.01,0.05}\right], \left[\mathrm{0.10,0.15}\right]\right)$
High (H)	$\left(\left[\mathrm{0.55,0.60}\right], \left[\mathrm{0.10,0.15}\right], \left[\mathrm{0.20,0.25}\right]\right)$
Medium (M)	$\left(\left[\mathrm{0.35,0.40}\right], \left[\mathrm{0.20,0.25}\right], \left[\mathrm{0.30,0.35}\right]\right)$
Low (L)	$\left(\left[\mathrm{0.15,0.20}\right], \left[\mathrm{0.30,0.35}\right], \left[\mathrm{0.40,0.45}\right]\right)$
Very Low (VL)	$\left(\left[\mathrm{0.01,0.01}\right], \left[\mathrm{0.40,0.44}\right], \left[\mathrm{0.50,0.55}\right]\right)$

*Step 3*: Apply the multiplicative AHP approach to determine the weight for each DM.

In this stage, we calculate the weight of each DM using the multiplicative AHP approach.

*Step 4*: Build the collective IVPF decision matrix.

To improve the GRP method in the process of group decision-making, it is essential to aggregate all individual decision matrices ${R}^{(k)}={\left({r}_{ij}^{(k)}\right)}_{m\times n}$ into the collective IVPF decision matrix $\widetilde{R}={\left({\widetilde{r}}_{ij}\right)}_{m\times n}$. This cluster is achieved through the application of the IVPFOWIA operator, as specified in Eq. (14):

22 $$\begin{array}{c}\widetilde{R}={\left({\widetilde{r}}_{ij}\right)}_{m\times n}=\left[\begin{array}{cccc}{\widetilde{r}}_{11}& {\widetilde{r}}_{12}& \cdots & {\widetilde{r}}_{1n}\\ {\widetilde{r}}_{21}& {\widetilde{r}}_{22}& \cdots & {\widetilde{r}}_{2n}\\ \vdots & \vdots & \vdots & \vdots \\ {\widetilde{r}}_{m1}& {\widetilde{r}}_{m2}& \cdots & {\widetilde{r}}_{mn}\end{array}\right] \end{array}$$

### Phase 2: Improve GRP method under IVPFS

Traditional GRP method is based on a single base point, and the similarity between the alternatives and the ideal solution is determined by calculating the cosine value of the angle between the alternatives and the ideal solution. Our research has improved the GRP method based on the existing literature by calculating the relative grey relation projection of each yacht design alternative based on the IVPFS theory as a way to select the optimal design alternative. The extended GRP method not only improves the accuracy of evaluation, but also enhances the rationality and effectiveness of decision-making. The specific steps of the improved GRP method are as follows:

*Step 1*: Normalize the decision-making evaluation matrix. In MCDM, we distinguish between two types of criteria: benefit type and cost type. Consequently, the risk evaluation matrix $\widetilde{R}={\left({\widetilde{r}}_{ij}\right)}_{m\times n}$ is transformed into a normalized decision matrix ${\widetilde{R}}^{*}={\left({\widetilde{r}}_{ij}^{*}\right)}_{m\times n}$. Where:

23 $$\begin{array}{*{20}c} {\tilde{r}_{ij}^{*} = \left\{ {\begin{array}{*{20}l} {r_{ij} ,} \hfill & {{\text{if}}\,C_{j} \,{\text{is }}\,{\text{a}}\,{\text{benefit - type}}\,{\text{criterion,}}} \hfill \\ {\overline{r}_{ij} ,} \hfill & {{\text{if}}\,C_{j} \,{\text{is }}\,{\text{cost - type}}\,{\text{criterion}}{.}} \hfill \\ \end{array} } \right.} \\ \end{array}$$

For $$i=\mathrm{1,2},\cdots m,j=\mathrm{1,2}\cdots ,n$$*.*

*Step 2*: Under the normalized evaluation decision matrix by Eq. (23).

(a) Determine the interval-valued picture fuzzy positive ideal solution (IVPF-PIS): ${{\text{R}}}^{+}$ can be obtained using Eq. (24):

24 $$\begin{array}{c}\left[{R}^{+}\right]=\left[{\widetilde{r}}_{1}^{+},{\widetilde{r}}_{2}^{+},{\widetilde{r}}_{3}^{+},\cdots ,{\widetilde{r}}_{n}^{+}\right] \end{array}$$

(b) Determine the interval-valued picture fuzzy negative ideal solution (IVPF-NIS), ${{\text{R}}}^{-}$ can be determined using Eq. (25):

25 $$\begin{array}{c}\left[{R}^{-}\right]=\left[{\widetilde{r}}_{1}^{-},{\widetilde{r}}_{2}^{-},{\widetilde{r}}_{3}^{-},\cdots ,{\widetilde{r}}_{n}^{-}\right] \end{array}$$

where

26 $$\begin{array}{c}{\widetilde{r}}_{j}^{+}=\left\{\begin{array}{c}{\mu }_{j}^{L+}=\underset{i}{max} {\mu }_{ij}^{L}\\ {\mu }_{j}^{U+}=\underset{i}{max} {\mu }_{ij}^{U}\\ {\eta }_{j}^{L+}=\underset{i}{min} {\eta }_{ij}^{L}\\ {\eta }_{j}^{U+}=\underset{i}{min} {\eta }_{ij}^{U}\\ {\nu }_{j}^{L+}=\underset{i}{min} {\nu }_{ij}^{L}\\ {\nu }_{j}^{U+}=\underset{i}{min} {\nu }_{ij}^{U}\end{array}\right. \end{array}$$

27 $$\begin{array}{c}{\widetilde{r}}_{j}^{-}=\left\{\begin{array}{c}{\mu }_{j}^{L-}=\underset{i}{min} {\mu }_{ij}^{L}\\ {\mu }_{j}^{U-}=\underset{i}{min} {\mu }_{ij}^{U}\\ {\eta }_{j}^{L-}=\underset{i}{min} {\eta }_{ij}^{L}\\ {\eta }_{j}^{U-}=\underset{i}{min} {\eta }_{ij}^{U}\\ {\nu }_{j}^{L-}=\underset{i}{max} {\nu }_{ij}^{L}\\ {\nu }_{j}^{U-}=\underset{i}{max} {\nu }_{ij}^{U}\end{array}\right. \end{array}$$

*Step 3*: Calculate positive and negative correlation matrices.

Represent the gray correlation matrix between the *i*th sample and the positive (negative) ideal sample as ${\varphi }^{+}$*(*${\varphi }^{-}$*),*where ${\varphi }_{ij}^{+} {\text{and}}$ ${\varphi }_{ij}^{-}$ are the individual elements:

28 $$\begin{array}{c}{\varphi }_{ij}^{+\left(-\right)}=\frac{{min}_{i}{min}_{j}d\left({\widetilde{r}}_{ij},{\widetilde{r}}_{j}^{+\left(-\right)}\right)+\rho {max}_{i}{max}_{j}d\left({\widetilde{r}}_{ij},{\widetilde{r}}_{j}^{+\left(-\right)}\right)}{d\left({\widetilde{r}}_{ij},{\widetilde{r}}_{j}^{+\left(-\right)}\right)+\rho {max}_{i}{max}_{j}d\left({\widetilde{r}}_{ij},{\widetilde{r}}_{j}^{+\left(-\right)}\right)} \end{array}$$

where $\rho$ is referred to as the resolution coefficient, serving to modify the scale of the comparison environment. $\rho =0$ implies the absence of a surrounding environment, while $\rho = 1$ signifies no alteration in the surrounding environment. Typically, $\rho = 0.5$. The term $d\left({\widetilde{r}}_{ij},{\widetilde{r}}_{j}^{+(-)}\right)$ represents the distance between ${\widetilde{r}}_{ij}$ and ${\widetilde{r}}_{j}^{+}({\widetilde{r}}_{j}^{-})$, calculable using Eq. (17).

Through the ${\varphi }_{ij}^{+\left(-\right)}\left(i=\mathrm{1,2},\cdots ,m,j=\mathrm{1,2},\cdots ,n\right)$, we can construct the two grey relational coefficient matrices:

29 $$\begin{array}{c}{\varphi }^{+}={\left({\varphi }_{ij}^{+}\right)}_{m\times n}=\left[\begin{array}{ccc}{\varphi }_{11}^{+}& {\varphi }_{12}^{+}& \begin{array}{cc}\cdots & {\varphi }_{1n}^{+}\end{array}\\ {\varphi }_{21}^{+}& {\varphi }_{22}^{+}& \begin{array}{cc}\cdots & {\varphi }_{2n}^{+}\end{array}\\ \begin{array}{c}\vdots \\ {\varphi }_{m1}^{+}\end{array}& \begin{array}{c}\vdots \\ {\varphi }_{m2}^{+}\end{array}& \begin{array}{c}\begin{array}{cc}\cdots & \vdots \end{array}\\ \begin{array}{cc}\cdots & {\varphi }_{mn}^{+}\end{array}\end{array}\end{array}\right]\end{array}$$

30 $$\begin{array}{c}{\mathrm{\varphi }}^{-}={\left({\mathrm{\varphi }}_{{\text{ij}}}^{-}\right)}_{{\text{m}}\times {\text{n}}}=\left[\begin{array}{ccc}{\mathrm{\varphi }}_{11}^{-}& {\mathrm{\varphi }}_{12}^{-}& \begin{array}{cc}\cdots & {\mathrm{\varphi }}_{1{\text{n}}}^{-}\end{array}\\ {\mathrm{\varphi }}_{21}^{-}& {\mathrm{\varphi }}_{22}^{-}& \begin{array}{cc}\cdots & {\mathrm{\varphi }}_{2{\text{n}}}^{-}\end{array}\\ \begin{array}{c}\vdots \\ {\mathrm{\varphi }}_{{\text{m}}1}^{-}\end{array}& \begin{array}{c}\vdots \\ {\mathrm{\varphi }}_{{\text{m}}2}^{-}\end{array}& \begin{array}{c}\begin{array}{cc}\cdots & \vdots \end{array}\\ \begin{array}{cc}\cdots & {\mathrm{\varphi }}_{{\text{mn}}}^{-}\end{array}\end{array}\end{array}\right]\end{array}$$

*Step 4*: Construct the two weighted grey relational coefficient matrices.

Two weighted grey relational coefficient matrices ${\psi }^{+}={\left({\psi }_{ij}^{+}\right)}_{m\times n}$ and ${\psi }^{-}={\left({\psi }_{ij}^{-}\right)}_{m\times n}$ can be calculated by Eqs. (31) and (32), respectively.

31 $$\begin{array}{c}{\psi }^{+}={\left({\psi }_{ij}^{+}\right)}_{m\times n}=\left[\begin{array}{ccc}{\psi }_{11}^{+}& {\psi }_{12}^{+}& \begin{array}{cc}\cdots & {\psi }_{1n}^{+}\end{array}\\ {\psi }_{21}^{+}& {\psi }_{22}^{+}& \begin{array}{cc}\cdots & {\psi }_{2n}^{+}\end{array}\\ \begin{array}{c}\vdots \\ {\psi }_{m1}^{+}\end{array}& \begin{array}{c}\vdots \\ {\psi }_{m2}^{+}\end{array}& \begin{array}{c}\begin{array}{cc}\cdots & \vdots \end{array}\\ \begin{array}{cc}\cdots & {\psi }_{mn}^{+}\end{array}\end{array}\end{array}\right] \end{array}$$

32 $$\begin{array}{c}{\psi }^{-}={\left({\psi }_{ij}^{-}\right)}_{m\times n}=\left[\begin{array}{ccc}{\psi }_{11}^{-}& {\psi }_{12}^{-}& \begin{array}{cc}\cdots & {\psi }_{1n}^{-}\end{array}\\ {\psi }_{21}^{-}& {\psi }_{22}^{-}& \begin{array}{cc}\cdots & {\psi }_{2n}^{-}\end{array}\\ \begin{array}{c}\vdots \\ {\psi }_{m1}^{-}\end{array}& \begin{array}{c}\vdots \\ {\psi }_{m2}^{-}\end{array}& \begin{array}{c}\begin{array}{cc}\cdots & \vdots \end{array}\\ \begin{array}{cc}\cdots & {\psi }_{mn}^{-}\end{array}\end{array}\end{array}\right] \end{array}$$

where ${\psi }_{ij}^{+}={w}_{j}{\varphi }_{ij}^{+}$, ${\psi }_{ij}^{-}={w}_{j}{\varphi }_{ij}^{-}$. ${w}_{j}$ is the weight of the criterion ${C}_{j}$, we can calculate it by Eqs. (18) and (19).

*Step 5*: Calculate the grey relational projections of each scheme ${A}_{i} (i = \mathrm{1,2},\dots ,m)$ on the IVPF-PIS and IVPF-NIS, respectively.

33 $$\begin{array}{*{20}l} {\tau _{i}^{ + } = \left\| {\varphi _{i}^{ + } } \right\| \times \cos \left( {\psi _{i}^{ + } ,\psi _{0}^{ + } } \right) = \frac{{\mathop \sum \nolimits_{{j = 1}}^{n} \left( {w_{j}^{2} \varphi _{{ij}}^{ + } } \right)}}{{\sqrt {\mathop \sum \nolimits_{{j = 1}}^{n} w_{j}^{2} } }},i = 1,2, \ldots ,m.} \\ \end{array}$$

34 $$\begin{array}{*{20}c} {\tau _{i}^{ - } = \left\| {\varphi _{i}^{ - } } \right\| \times \cos \left( {\psi _{i}^{ - } ,\psi _{0}^{ - } } \right) = \frac{{\mathop \sum \nolimits_{{j = 1}}^{n} \left( {w_{j}^{2} \varphi _{{ij}}^{ - } } \right)}}{{\sqrt {\mathop \sum \nolimits_{{j = 1}}^{n} w_{j}^{2} } }},i = 1,2, \ldots ,m.} \\ \end{array}$$

### Phase 3: Sort according to the final results and select the best design scheme

The relative grey relational projection of every alternative to the IVPF-PIS ${\psi }_{0}^{+}=\left({w}_{1},{w}_{2},\ldots ,{w}_{n}\right)$ is defined as follows:

35 $$\begin{array}{c}{\tau }_{i}=\frac{{\tau }_{i}^{+}}{{{\tau }_{i}^{+}+\tau }_{i}^{-}} i=\mathrm{1,2},\ldots ,m. \end{array}$$

The results are arranged in ascending order based on the values of ${\tau }_{i}$. The relative closeness ${\tau }_{i}$ signifies the proximity of scheme ${A}_{i}$ to the ideal scheme. As the relative closeness become greater, the scheme improves.

### Ethical approval

This article does not contain any studies with human participants or animals performed by any of the authors.

## Case study

### Choosing the optimal alternative with the proposed methodology

In this phase, the aforementioned approach is employed to identify the optimal design among yacht alternatives. All DMs are seasoned experts in yacht design, possessing extensive design expertise. These DMs constitute an evaluation and selection group, comprising 10 members denoted as $D=\left\{{D}_{1},{D}_{2},\ldots ,{D}_{10}\right\}$, and considering three concept design alternatives $A=\left\{{A}_{1},{A}_{2},{A}_{3}\right\}$. The data, assessed by the 10 DMs, is represented as IVPFNs after statistical processing. Refer to the table below for the decision-making information. Following the outlined procedures of the proposed model, the specific steps for design concept evaluation are detailed as follows:

### Phase 1: Construct the collective IVPF decision matrix

*Step 1*: Determine the evaluation index evaluation system of design concept by the Kano model. First, we analyze the data through questionnaires, and the initial CRs for yacht design were determined as shown in Table 9.

**Table 9:** Collection of the initial CRs for yacht design concept schemes.

Emphasize uniqueness and eye-catching features	Allow adjustable cabin layouts for versatility	Use efficient engines and sustainable technologies
Design comfortable living spaces	Create a unique water activity platform	Advanced smart home systems
Ensure private social spaces	Provide customized services	Provide emergency response systems
Integrate renewable energy sources	Ensure robust connectivity for entertainment	Design efficient storage solutions

During Kano model evaluation on the attribute set shown in Table 9, 126 questionnaires were issued and returned, including 120 valid results. The statistical results are shown in Table 10.

**Table 10:** Analysis results of quality factors of yacht product design.

Functional requirements	A	O	M	I	R	Q	Attributes classification
Provide customized services	50	35	30	2	3	0	A
Allow adjustable cabin layouts for versatility	10	16	56	30	8	0	M
Use efficient engines and sustainable technologies	30	34	7	46	3	0	I
Create a unique water activity platform	9	24	31	55	0	1	I
Advanced smart home systems	55	40	10	5	10	0	A
Provide emergency response systems	11	58	7	35	7	1	O
Design efficient storage solutions	8	43	10	47	12	0	I
Emphasize uniqueness and eye-catching features	15	38	47	13	6	1	M
Design comfortable living spaces	47	28	36	7	2	0	A
Integrate renewable energy sources	54	35	11	10	10	0	A
Ensure robust connectivity for entertainment	12	20	36	44	8	0	I
Ensure private social spaces	16	46	24	34	0	0	O

*Q* questionable, *A* attractive, *O* one-dimensional, *M* must-be, *I* indifference, *R*: reverse.

**Figure 4:**
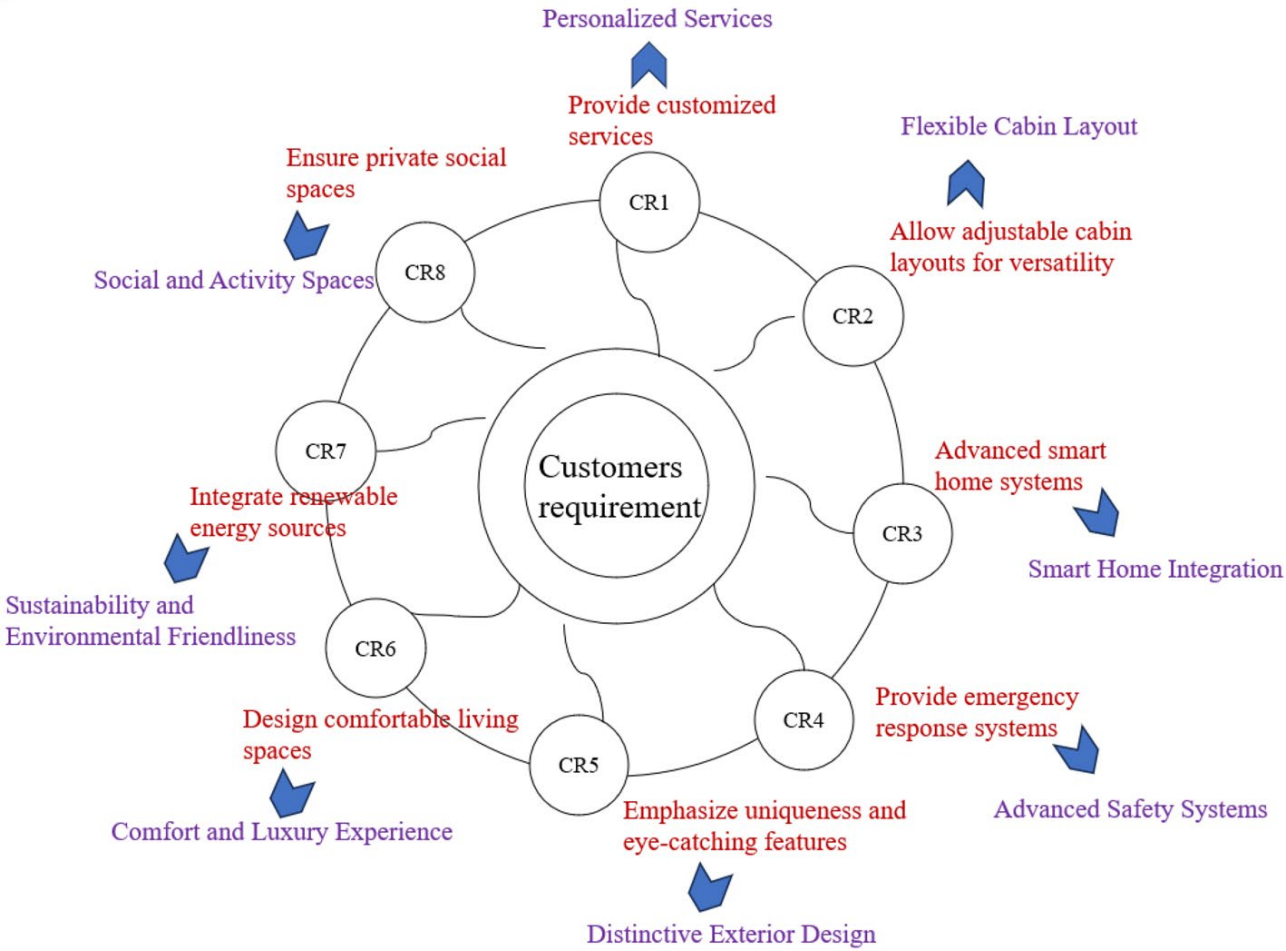
The mapping relation of CRs- design concept evaluation index.

According to Kano’s customer satisfaction model, the fundamental elements with A/M/O attributes are considered core requirements. By utilizing the mapping relationship shown in Fig. 2, CRs are translated into evaluation criteria for the assessment of design concepts, as illustrated in Fig. 4. It is crucial to understand that there is a unique, one-to-one correspondence in this mapping process.

*Step 2*: Construct the IVPF decision matrix for each DM.

Taking DM ${{\text{R}}}^{1}$ for example, the decision matrix for DM ${{\text{R}}}^{1}$ is built as shown in Table 11. And all the DMs evaluated three yachts design alternatives $A=\left\{{A}_{1},{A}_{2},{A}_{3}\right\}$ according to the attributes, as shown in Appendix A.

**Table 11:** Linguistic evaluations of preference matrix for DM ${{\text{R}}}^{1}$.

	A_1_	A_2_	A_3_
${{\text{C}}}_{1}$	H	VH	H
${{\text{C}}}_{2}$	L	VH	VH
${{\text{C}}}_{3}$	H	H	VH
${{\text{C}}}_{4}$	H	H	M
${{\text{C}}}_{5}$	VH	M	H
${{\text{C}}}_{6}$	L	H	VH
${{\text{C}}}_{7}$	H	M	L
${{\text{C}}}_{8}$	L	L	M

The linguistic evaluation value matrix in Table 8 can be converted into an IVPFN matrix through Table 11, as shown in Table 12.

**Table 12:** The IVPF matrix of DM1.

	A_1_	A_2_	A_3_
${{\text{C}}}_{1}$	($\left[.55,.60\right],\left[.10,.15\right],\left[.20,.25\right]$)	($\left[.75,.80\right],\left[.01,.05\right],\left[.10,.15\right]$)	($\left[.55,.60\right],\left[.10,.15\right],\left[.20,.25\right]$)
${{\text{C}}}_{2}$	($\left[.15,.20\right],\left[.30,.35\right],\left[.40,.45\right]$)	($\left[.75,.80\right],\left[.01,.05\right],\left[.10,.15\right]$)	($\left[.75,.80\right],\left[.01,.05\right],\left[.10,.15\right]$)
${{\text{C}}}_{3}$	($\left[.55,.60\right],\left[.10,.15\right],\left[.20,.25\right]$)	($\left[.55,.60\right],\left[.10,.15\right],\left[.20,.25\right]$)	($\left[.75,.80\right],\left[.01,.05\right],\left[.10,.15\right]$)
${{\text{C}}}_{4}$	($\left[.55,.60\right],\left[.10,.15\right],\left[.20,.25\right]$)	($\left[.55,.60\right],\left[.10,.15\right],\left[.20,.25\right]$)	($\left[.35,.40\right],\left[.20,.25\right],\left[.30,.35\right]$)
${{\text{C}}}_{5}$	($\left[.75,.80\right],\left[.01,.05\right],\left[.10,.15\right]$)	($\left[.35,.40\right],\left[.20,.25\right],\left[.30,.35\right]$)	($\left[.55,.60\right],\left[.10,.15\right],\left[.20,.25\right]$)
${{\text{C}}}_{6}$	($\left[.15,.20\right],\left[.30,.35\right],\left[.40,.45\right]$)	($\left[.55,.60\right],\left[.10,.15\right],\left[.20,.25\right]$)	($\left[.75,.80\right],\left[.01,.05\right],\left[.10,.15\right]$)
${{\text{C}}}_{7}$	($\left[.55,.60\right],\left[.10,.15\right],\left[.20,.25\right]$)	($\left[.35,.40\right],\left[.20,.25\right],\left[.30,.35\right]$)	($\left[.15,.20\right],\left[.30,.35\right],\left[.40,.45\right]$)
${{\text{C}}}_{8}$	($\left[.15,.20\right],\left[.30,.35\right],\left[.40,.45\right]$)	($\left[.15,.20\right],\left[.30,.35\right],\left[.40,.45\right]$)	($\left[.35,.40\right],\left[.20,.25\right],\left[.30,.35\right]$)

Where: take A_1_-C_1_ as an example, ($\left[.55,.60\right],\left[.\mathrm{10,0.15}\right],\left[.20,.25\right]$) is the abbreviation of ($\left[0.\mathrm{55,0.60}\right],\left[0.\mathrm{10,0.15}\right],\left[0.\mathrm{20,0.25}\right]$).

*Step 3*: Determine the weights of DMs by the multiplicative AHP approach.

With the help of the multiplicative AHP approach, we compute the weights of DMs $\omega ={\left({\omega }_{1},{\omega }_{2},\dots ,{\omega }_{10}\right)}^{T}={\left(\mathrm{0.213,0.213,0.213,0.0533,0.0533,0.0533,0.0503,0.0503,0.0503,0.0503}\right)}^{T}$

*Step 4*: Construct the collective IVPF decision matrix.

Through the application of the IVPFOWIA, the collective decision matrix is derived, as depicted in Table 13.

**Table 13:** The collective decision matrix.

	A_1_	A_2_	A_3_
${{\text{C}}}_{1}$	($\left[.54,.59\right],\left[.10,.15\right],\left[.20,.25\right]$)	($\left[.61,.66\right],\left[.07,.12\right],\left[.17,.22\right]$)	($\left[.60,.65\right],\left[.08,.12\right],\left[.18,.23\right]$)
${{\text{C}}}_{2}$	($\left[.49,.54\right],\left[.13,.17\right],\left[.24,.29\right]$)	($\left[.58,.64\right],\left[.08,.13\right],\left[.19,.24\right]$)	($\left[.61,.66\right],\left[.07,.11\right],\left[.17,.22\right]$)
${{\text{C}}}_{3}$	($\left[.53,.58\right],\left[.11,.16\right],\left[.21,.26\right]$)	($\left[.52,.57\right],\left[.11,.16\right],\left[.22,.27\right]$)	($\left[.64,.69\right],\left[.06,.10\right],\left[.16,.21\right]$)
${{\text{C}}}_{4}$	($\left[.54,.59\right],\left[.10,.15\right],\left[.21,.26\right]$)	($\left[.47,.52\right],\left[.14,.19\right],\left[.24,.29\right]$)	($\left[.37,.42\right],\left[.19,.24\right],\left[.29,.34\right]$)
${{\text{C}}}_{5}$	($\left[.64,.69\right],\left[.06,.10\right],\left[.16,.21\right]$)	($\left[.39,.44\right],\left[.18,.23\right],\left[.28,.33\right]$)	($\left[.53,.58\right],\left[.11,.16\right],\left[.21,.26\right]$)
${{\text{C}}}_{6}$	($\left[.28,.33\right],\left[.23,.28\right],\left[.33,.38\right]$)	($\left[.61,.67\right],\left[.06,.11\right],\left[.17,.21\right]$)	($\left[.65,.71\right],\left[.05,.09\right],\left[.15,.20\right]$)
${{\text{C}}}_{7}$	($\left[.45,.51\right],\left[.15,.19\right],\left[.25,.30\right]$)	($\left[.36,.42\right],\left[.19,.24\right],\left[.29,.34\right]$)	($\left[.23,.29\right],\left[.25,.30\right],\left[.36,.41\right]$)
${{\text{C}}}_{8}$	($\left[.25,.30\right],\left[.25,.30\right],\left[.35,.40\right]$)	($\left[.37,.43\right],\left[.18,.23\right],\left[.29,.34\right]$)	($\left[.46,.51\right],\left[.14,.19\right],\left[.24,.29\right]$)

Where: take A_1_-C_1_ as an example, ($\left[.54,.59\right],\left[.10,.15\right],\left[.20,.25\right]$) is the abbreviation of ($\left[0.\mathrm{54,0.59}\right],\left[0.\mathrm{10,0.15}\right],\left[0.\mathrm{20,0.25}\right]$).

*Step 5*: With the help of Eqs. (18)–(19), we can determine the entropy weights of IVPFS of $C=\left\{{C}_{1},{C}_{2},{C}_{3},{C}_{4},{C}_{5},{C}_{6},{C}_{7},{C}_{8}\right\}$ is $w={\left(\mathrm{0.167,0.133,0.37,0.048,0.119,0.223,0.090,0.082}\right)}^{T}$ .

### Phase 2: Improved GRP method under IVPFS

*Step 1*: Given that all eight criteria are benefits (not costs), according to Eq. (23), the standardized evaluation decision matrix aligns with the contents of Table 13.

*Step 2*: The IVPF-PIS and IVPF-NIS of the collective decision matrix are calculated through Eqs. (24)–(25).

$$\begin{aligned} R^{ + } & = \{ \left( {\left[ {0.61,0.66} \right],\left[ {0.07,0.12} \right],\left[ {0.17,0.22} \right]} \right),\left( {\left[ {0.61,0.66} \right],\left[ {0.07,0.12} \right],\left[ {0.17,0.22} \right]} \right), \left( {\left[ {0.64,0.69} \right],\left[ {0.06,0.10} \right],\left[ {0.16,0.21} \right]} \right) \\ & \left( {\left[ {0.54,0.59} \right],\left[ {0.10,0.15} \right],\left[ {0.21,0.26} \right]} \right),\left( {\left[ {0.64,0.69} \right],\left[ {0.06,0.10} \right],\left[ {0.16,0.21} \right]} \right),\left( {\left[ {0.65,0.71} \right],\left[ {0.05,0.09} \right],\left[ {0.15,0.20} \right]} \right) \\ & \left( {\left[ {0.45,0.51} \right],\left[ {0.15,0.19} \right],\left[ {0.25,0.30} \right]} \right),\left( {\left[ {0.46,0.51} \right],\left[ {0.14,0.19} \right],\left[ {0.24,0.29} \right]} \right)\} \\ \end{aligned}$$

$$\begin{aligned} {\text{R}}^{ - } & = \{ \left( {\left[ {0.54,0.59} \right],\left[ {0.10,0.15} \right],\left[ {0.20,0.25} \right]} \right),\left( {\left[ {0.49,0.54} \right],\left[ {0.10,0.17} \right],\left[ {0.24,0.29} \right]} \right),{\text{~}}\left( {\left[ {0.54,0.57} \right],\left[ {0.11,0.16} \right],\left[ {0.22,0.27} \right]} \right) \\ & \quad \left( {\left[ {0.37,0.42} \right],\left[ {0.19,0.24} \right],\left[ {0.29,0.34} \right]} \right),\left( {\left[ {0.39,0.44} \right],\left[ {0.18,0.23} \right],\left[ {0.28,0.33} \right]} \right),\left( {\left[ {0.28,0.33} \right],\left[ {0.23,0.28} \right],\left[ {0.33,0.38} \right]} \right) \\ & \quad \left( {\left[ {0.23,0.29} \right],\left[ {0.25,0.30} \right],\left[ {0.36,0.41} \right]} \right),\left( {\left[ {0.25,0.30} \right],\left[ {0.25,0.30} \right],\left[ {0.35,0.40} \right]} \right)\} \\ \end{aligned}$$

*Step 3*: Determine the grey relational coefficient matrices by Eqs. (29) and (30).

$${{\varphi }_{ij}^{+}}_{3\times 8}=\left[\begin{array}{cccccccc}0.74& 0.59& 0.63& 1.00& 1.00& 0.33& 1.00& 0.46\\ 1.00& 0.86& 0.61& 0.72& 0.42& 0.84& 0.67& 0.67\\ 0.93& 1.00& 1.00& 0.52& 0.63& 1.00& 0.46& 1.00\end{array}\right]$$

$${{\varphi }_{ij}^{-}}_{3\times 8}=\left[\begin{array}{cccccccc}1.00& 1.00& 0.94& 0.52& 0.43& 1.00& 0.46& 1.00\\ 0.74& 0.67& 1.00& 0.65& 1.00& 0.36& 0.59& 0.60\\ 0.78& 0.60& 0.61& 1.00& 0.57& 0.33& 1.00& 0.46\end{array}\right]$$

*Step 4*: Calculate the weighted grey relational coefficient matrices through Eqs. (31) and (32), respectively.

$${{\psi }_{ij}^{+}}_{3\times 8}=\left[\begin{array}{cccccccc}0.12& 0.08& 0.09& 0.05& 0.12& 0.07& 0.09& 0.04\\ 0.17& 0.11& 0.09& 0.04& 0.05& 0.18& 0.06& 0.05\\ 0.16& 013& 0.14& 0.03& 0.08& 0.22& 0.04& 0.08\end{array}\right]$$

$${{\psi }_{ij}^{-}}_{3\times 8}=\left[\begin{array}{cccccccc}0.17& 0.13& 0.13& 0.02& 0.05& 0.22& 0.04& 0.08\\ 0.13& 0.09& 0.14& 0.03& 0.12& 0.08& 0.05& 0.05\\ 0.13& 0.07& 0.08& 0.05& 0.07& 0.07& 0.09& 0.04\end{array}\right]$$

### Phase 3: Sort according to the final results and select the best design scheme

Compute the grey relational projections of each alternative ${A}_{i} (i = \mathrm{1,2},3)$ on the IVPF-PIS and IVPF-NIS through Eqs. (33)–(35), respectively. The detailed parameters and alternatives are provided in Table 14.

**Table 14:** The order of the three alternatives.

	${\tau }_{i}^{+}$	${\tau }_{i}^{-}$	IVPFS-Improved GRP method	
			${\tau }_{i}$	Rank
A_1_	0.230	0.342	0.402	3
A_2_	0.298	0.278	0.546	2
A_3_	0.347	0.216	0.616	1

According to the ${\tau }_{i}$, the ranking order is A_3_ ≻ A_2_ ≻ A_1_.

### Sensitivity analysis

**Figure 5:**
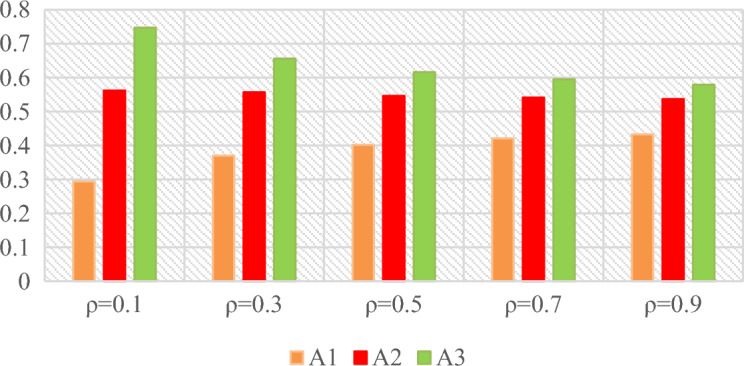
Sensitivity analysis by different resolution coefficient $\rho$.

In this section, in order to further investigate the evaluation process of the IVPF-improved GRP method, a sensitivity analysis of the resolution coefficient $\rho$ was conducted. When $\rho =0.5$, the ranking of the three design concept alternatives is A_3_ ≻ A_2_ ≻ A_1_. Table 15 shows the ${\tau }_{i}$ for different resolution coefficients $\rho$, and the corresponding figures are shown in Fig. 5. As shown in Fig. 5, A3 is consistently the optimal choice among the three design concept alternatives. It can be observed from Fig. 5 that as the resolution coefficient $\rho$ changes, the gap between alternative 2 and alternative 3 gradually narrows. However, the ranking of the design concept alternatives remains unchanged (A_3_ ≻ A_2_ ≻ A_1_). Therefore, the proposed improved GRP method based on IVPFS demonstrates stability and reliability in the evaluation of design concept alternatives.

**Table 15:** The ${\tau }_{i}$ of design concept alternatives with different resolution coefficient $\rho$.

	A_1_	A_2_	A_3_
$\rho =0.1$	0.294	0.562	0.747
$\rho =0.3$	0.370	0.557	0.656
$\rho =0.5$	0.402	0.546	0.616
$\rho =0.7$	0.421	0.541	0.595
$\rho =0.9$	0.433	0.537	0.579

Alternatively, sensitivity analysis allows for a variety of change techniques. Because of space constraints, this research has only included the examples where the resolution coefficient $\rho$ is employed. More extensions can be added to improve sensitivity analysis in the future research.

### Comparative analysis and discussion

**Figure 6:**
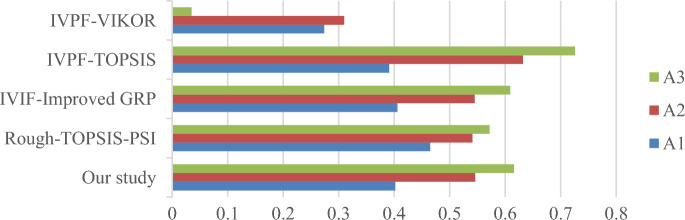
The close index between the four MAGDM methods.

To assess the effectiveness of the proposed methodology, comparative studies are conducted alongside the case study, utilizing the Rough Entropy TOPSIS-PSI method^52^, Interval-Valued Intuitionistic Fuzzy (IVIF)-Improved GRP method, IVPF-VIKOR method^53^ and IVPF-TOPSIS method. Table 16 and Fig. 6 present the results of a comprehensive comparison among different methodologies.

**Table 16:** The results of comparisons between different methods.

Design concepts	Rough-TOPSIS-PSI		IVIF-Improved GRP		IVPF-TOPSIS	
	${CI}_{i}$	Rank	${\delta }_{i}$	Rank	${\xi }_{i}$	Rank
A_1_	0.465	3	0.406	3	0.391	3
A_2_	0.541	2	0.545	2	0.632	2
A_3_	0.572	1	0.609	1	0.726	1

Design concepts	IVPF-VIKOR		Proposal method			
	${Q}_{i}$	Rank	${\tau }_{i}$	Rank		
A_1_	0.274	2	0.402	3		
A_2_	0.310	3	0.546	2		
A_3_	0.035	1	0.616	1

From Fig. 6 it can be seen that ${A}_{3}$ represents the best alternative for yacht design through the Rough Entropy TOPSIS-PSI, IVPF-improved GRP, IVPF-VIKOR and IVPF-TOPSIS. From Fig. 6, it can be seen that there are certain differences between different optimization models. These differences are reflected in the entire design optimization process or certain data processing stages. The specific details are summarized as follows:

1. Rough Entropy TOPSIS-PSI method: it is proposed by Chen, this method is fundamentally rooted in rough sets. The ranking approach emphasizes the subjectivity of the data, establishes a fuzzy environment using rough numbers, and finalizes scheme selection through proximity coefficients based on the TOPSIS method. Notably, this method does not consider DMs weights in the calculation process. Additionally, an interval weight calculation method based on entropy weight in the form of intervals is introduced for attribute weight calculation.
2. IVIF- Improved GRP method: The main difference between this method and our model is the fuzzy environment used. As a method based on IVIFS, the IVIF-Improved GRP method has been successful in applications, but as an extended form of interval fuzzy sets, it does not take into account the degree of neutral when describing uncertain information compared to IVPFS, which means that IVIFS are not as detailed as IVPFS when describing uncertainty. As detailed and accurate as the IVPFS.
3. IVPF-TOPSIS method: The IVPF-TOPSIS method differs from our proposed model in the ranking model; the IVPF-TOPSIS method ranks the alternatives based on relative proximity. This method may be computationally more time-consuming, especially when dealing with a large amount of data or multiple attributes, and is unable to focus on the trends and similarities of the data sequences, leading to inaccurate final ranking results.
4. IVPF-VIKOR method: In this method, uncertainty and ambiguity in the decision-making process are addressed due to the benefits of the IVPFS environment. VIKOR method is used to reflect multiple criteria inherited from the selection problem into the solution, however, the VIKOR method may be affected by outliers, which may lead to unstable decision results in the presence of extreme values or outliers. of instability in the presence of extreme values or outliers.

**Figure 7:**
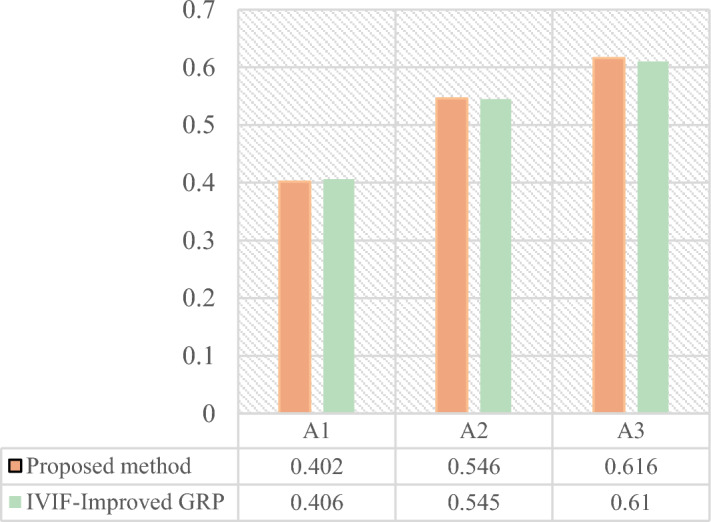
The Close Index between the two MAGDM methods.

The comparison with the Rough Entropy TOPSIS-PSI method is presented in Table 17. Despite certain dissimilarities between the two methods, they share a foundation in membership relationships and linguistic information. Ultimately, both approaches apply a compromise theory-based model for design concept scheme optimization and ranking. Additionally, the grey correlation projection value ${\tau }_{i}$ involved in our method bears similarity to the calculation form of the closeness coefficient ${CI}_{i}$ in the Rough Entropy TOPSIS-PSI method. The values of both exhibit a positive relationship within the interval [0,1]. Consequently, ${\tau }_{i}$ and ${CI}_{i}$ are compared, as depicted in Fig. 7. The results indicate that the scheme ranking of the Rough Entropy TOPSIS-PSI method aligns with the method based on membership relationships proposed in this manuscript. In both cases, ${A}_{3}>{A}_{2}>{A}_{1}$, signifying that ${A}_{3}$ is the optimal design concept scheme. Notably, the differentiation between the three schemes in the method introduced in this chapter is more pronounced, showcasing a greater level of distinction compared to the Rough Entropy TOPSIS-PSI method.

**Table 17:** Values of the coefficients and the rankings of the alternatives based on Rough-TOPSIS-PSI.

Design concept alternative	${A}_{1}$	${A}_{2}$	${A}_{3}$
${D}_{Pi}^{*}$	5.361	4.829	4.019
${D}_{Ni}^{*}$	4.654	5.700	5.373
${CI}_{i}$	0.465	0.541	0.572
Ranking	3	2	1

**Figure 8:**
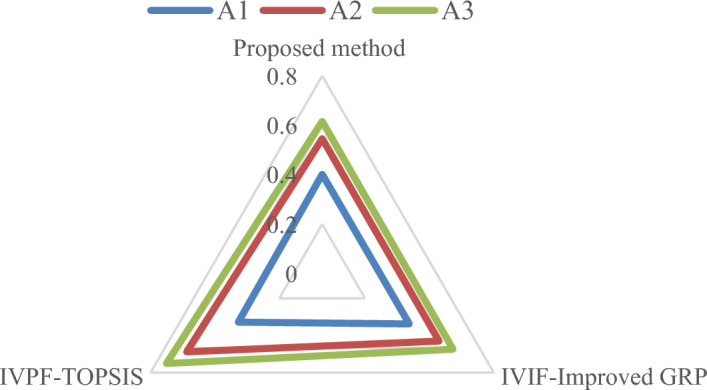
The comparison among the proposed method and IVIF-Improved GRP and IVPF-TOPSIS.

Figure 8 presents a comparison between the method proposed in this paper, the IVIF-Improved GRP method, and the IVPF-TOPSIS method. The results of the method proposed in this study and the IVIF-Improved GRP method exhibit similarities. In comparison with the IVIF-Improved GRP method, our proposed model possesses distinct advantages in addressing MADM problems. As an extension of IVIFS, IVPFS incorporate an increased neutral membership degree, providing richer decision information and aligning more closely with human cognition.

Furthermore, the IVPF-TOPSIS method differs from the above two methods in the ranking model, leading to some variations in the results. However, the ranking among the schemes has not undergone significant changes. Consequently, we assert that our IVPF-Improved GRP approach, as proposed in this manuscript, is more reliable and accurate in decision-making processes.

**Figure 9:**
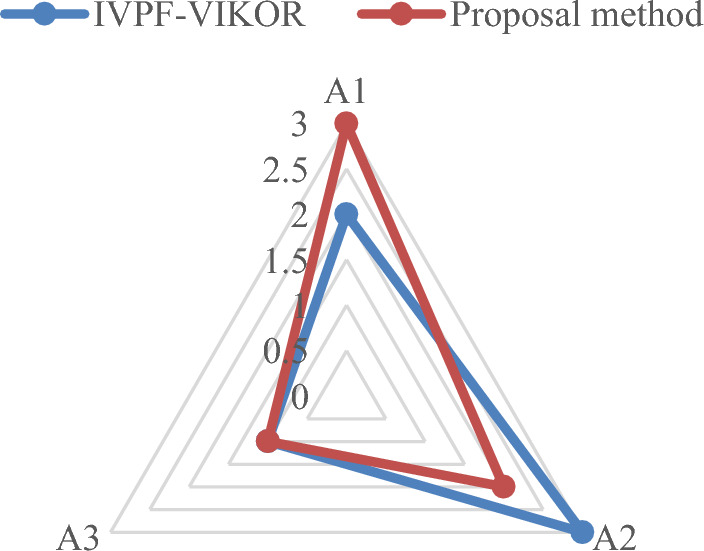
The comparison among the proposed method and IVPF-VIKOR method.

The comparison of the method proposed in this study with the IVPF-VIKOR method is shown in Fig. 9. From Fig. 9 it can be seen that ${A}_{3}$ is the best design concept alternative. However, except for alternative 3, which is consistent, there are some differences in the other ranking results of the two models. One reason for this is because each attribute is not independent of the other during the design concept evaluation process. Although the internal relationship is not clear, there is actually some correlation. the VIKOR method cannot handle the correlation between the indicators internally; the second reason is that when the attributes have discrete sample data, the improved GRP method can avoid the unilateral bias, which is the bias resulting from comparing a single attribute for each alternative, and thus comprehensively analyze the relationship between the criteria, reflecting the impact of the whole attribute space.

Ultimately, the improved GRP approach with IVPF can be adjusted to accommodate any quantity of alternatives, evaluation criteria, resulting in a minimal increase in its complexity. Consequently, this expanded version of the GRP method is applicable to addressing any MCDM issue within the context of IVPFS.

## Conclusion

The evaluation of design concepts plays a crucial role in the product development process. The purpose of this study is to introduce an innovative approach for design concept evaluation, taking into account inherent ambiguity and uncertainty present in information. The main contributions of this research are summarized as follows:

i. Utilizing the Kano model, the mapping relation between CRs and the evaluation index, we construct the decision attributes set for the design concept evaluation.
ii. By applying IVPFS theory, this research effectively identifies and characterizes ambiguity and uncertainty in design concept evaluation. Specifically, we adopt a practical approach, transforming linguistic information in concept design evaluation into IVPFNs, facilitating flexible decision-making procedures.
iii. Enhancements to the GRP method leads to the construction of IVPF-PIS and IVPF-NIS. The distance relationship between each scheme and IVPF-PIS and IVPF-NIS is calculated, ultimately determining the optimal design concept scheme by comparing the relative grey relational projection of each scheme. This improvement avoids the problem of inaccurate results caused by traditional GRP methods based on calculations from a single base point.

Results from a real yacht design case demonstrate the success of our proposed method in addressing the challenges of evaluating product conceptual designs in uncertain and ambiguous environments. It was compared with the Rough Entropy TOPSIS-PSI, IVPF-improved GRP, IVPF-VIKOR and IVPF-TOPSIS method. The results also showed that this novel method can effectively evaluate product concept design schemes.

Furthermore, our research lays the groundwork for potential future outcomes, such as applications in green supply chain management, project ranking, urban planning, and environmental governance. Future studies also can further explore the applicability and effectiveness of this framework across different industries and decision-making contexts, as well as how to further optimize the model for broader applications.

### Supplementary Information

Supplementary Information.

## References

[1] Qi J, Hu J, Peng Y (2021). Modified rough VIKOR based design concept evaluation method compatible with objective design and subjective preference factors. Appl. Soft Comput..

[2] Sun HY, Ma Q, Chen Z, Si GY (2023). A novel decision-making approach for product design evaluation using improved TOPSIS and GRP method under picture fuzzy set. Int. J. Fuzzy Syst..

[3] Dou YB (2023). A concept evaluation approach based on incomplete information: Considering large-scale criteria and risk attitudes. Adv. Eng. Inform..

[4] Li J, Shao Y, Qi X (2021). On variable-precision-based rough set approach to incomplete interval-valued fuzzy information systems and its applications. J. Intell. Fuzzy Syst. Appl. Eng. Technol..

[5] Shidpour H, Da Cunha C, Bernard A (2016). Group multi-criteria design concept evaluation using combined rough set theory and fuzzy set theory. Expert Syst. Appl..

[6] Zadeh LA (1965). Fuzzy sets. Inf. Control.

[7] Atanassov K, Vassilev P (2020). Intuitionistic fuzzy sets and other fuzzy sets extensions representable by them. J. Intell. Fuzzy Syst..

[8] Torra V (2010). Hesitant fuzzy sets. Int. J. Intell. Syst..

[9] Luo M, Sun Z, Xu D, Wu L (2024). Fuzzy inference full implication method based on single valued neutrosophic t-representable t-norm: Purposes, strategies, and a proof-of-principle study. Neutrosophic Syst. Appl..

[10] Mohamed A, Mohammed J, Sameh SA (2023). A neutrosophic framework for assessment of distributed circular water to give neighborhoods analysis to prepare for unexpected stressor events. Neutrosophic Syst. Appl..

[11] Ganie AH, Singh S, Khalaf MM, Al-Shamiri MMA (2022). On some measures of similarity and entropy for Pythagorean fuzzy sets with their applications. Comput. Appl. Math..

[12] Cuong, B. C., Kreinovich, V. & Ieee. In *Third World Congress on Information and Communication Technologies (WICT).* pp. 1–6.

[13] Kano (1984). Attractive quality and must-be quality. J. Jpn. Soc. Qual. Control.

[14] Shang B, Chen Z, Ma Q, Tan YH (2023). A comprehensive mortise and tenon structure selection method based on Pugh’s controlled convergence and rough Z-number MABAC method. PLoS ONE.

[15] Wu CT, Wang MT, Liu NT, Pan TS (2015). Developing a Kano-based evaluation model for innovation design. Math. Probl. Eng..

[16] Jin J, Jia DP, Chen KJ (2022). Mining online reviews with a Kansei-integrated Kano model for innovative product design. Int. J. Prod. Res..

[17] Zhu GN, Hu J, Ren HL (2020). A fuzzy rough number-based AHP-TOPSIS for design concept evaluation under uncertain environments. Appl. Soft Comput..

[18] Jiang C, Han X, Li D (2012). A new interval comparison relation and application in interval number programming for uncertain problems. Cmc-Comput. Mater. Contin..

[19] Yao N, Ye Y, Wang Q, Hu N (2020). Interval number ranking method considering multiple decision attitudes. Iran. J. Fuzzy Syst..

[20] Caichuan W, Jiajun L, Hasmat M, Gopal C, Smriti S (2022). Project investment decision based on VIKOR interval intuitionistic fuzzy set. J. Intell. Fuzzy Syst..

[21] Zeng S, Llopis-Albert C, Zhang Y (2018). A novel induced aggregation method for intuitionistic fuzzy set and its application in multiple attribute group decision making. Int. J. Intell. Syst..

[22] Kahraman C (2024). Proportional picture fuzzy sets and their AHP extension: Application to waste disposal site selection. Expert Syst. Appl..

[23] Luo MX, Zhang GF (2023). Divergence-based distance for picture fuzzy sets and its application to multi-attribute decision-making. Soft Comput..

[24] Wang T, Wu XX, Garg H, Liu Q, Chen GR (2023). A prospect theory-based MABAC algorithm with novel similarity measures and interactional operations for picture fuzzy sets and its applications. Eng. Appl. Artif. Intell..

[25] Naeem M, Qiyas M, Abdullah S (2021). An approach of interval-valued picture fuzzy uncertain linguistic aggregation operator and their application on supplier selection decision-making in logistics service value concretion. Math. Probl. Eng..

[26] Khalil AM, Li SG, Garg H, Li H, Ma S (2019). New operations on interval-valued picture fuzzy set, interval-valued picture fuzzy soft set and their applications. IEEE Access.

[27] Mishra AR, Rani P, Alrasheedi AF, Dwivedi R (2023). Evaluating the blockchain-based healthcare supply chain using interval-valued Pythagorean fuzzy entropy-based decision support system. Eng. Appl. Artif. Intell..

[28] Hua Z, Jing XC (2023). A generalized Shapley index-based interval-valued Pythagorean fuzzy PROMETHEE method for group decision-making. Soft Comput..

[29] Cao G, Shen LX (2023). A novel parameter similarity measure between interval-valued picture fuzzy sets with its application in pattern recognition. J. Intell. Fuzzy Syst..

[30] Mahmood T, Waqas HM, Ali Z, Ullah K, Pamucar D (2021). Frank aggregation operators and analytic hierarchy process based on interval-valued picture fuzzy sets and their applications. Int. J. Intell. Syst..

[31] Zhang D, Hu JH (2024). A novel multi-interval-valued fuzzy set model to solve MADM problems. Expert Syst. Appl..

[32] Büyüközkan G, Göçer F (2017). Application of a new combined intuitionistic fuzzy MCDM approach based on axiomatic design methodology for the supplier selection problem. Appl. Soft Comput..

[33] Jing LT (2021). A rough set-based interval-valued intuitionistic fuzzy conceptual design decision approach with considering diverse customer preference distribution. Adv. Eng. Inform..

[34] Singh A, Kumar S (2021). Picture fuzzy set and quality function deployment approach based novel framework for multi-criteria group decision making method. Eng. Appl. Artif. Intell..

[35] Kahraman C, Oztaysi B, Onar S (2022). A novel interval valued picture fuzzy TOPSIS method: Application on supplier selection. J. Mult.-Valued Logic Soft Comput..

[36] Akay D, Kulak O, Henson B (2011). Conceptual design evaluation using interval type-2 fuzzy information axiom. Comput. Ind..

[37] Zhu G-N, Hu J, Qi J, Gu C-C, Peng Y-H (2015). An integrated AHP and VIKOR for design concept evaluation based on rough number. Adv. Eng. Inform..

[38] Aikhuele D, Turan F (2017). An integrated fuzzy dephi and interval-valued intuitionistic fuzzy M-Topsis model for design concept selection. Pak. J. Stat. Oper. Res..

[39] Tiwari V, Jain PK, Tandon P (2017). An integrated Shannon entropy and TOPSIS for product design concept evaluation based on bijective soft set. J. Intell. Manuf..

[40] Hayat K, Ali MI, Karaaslan F, Cao BY, Shah MH (2020). Design concept evaluation using soft sets based on acceptable and satisfactory levels: An integrated TOPSIS and Shannon entropy. Soft Comput..

[41] Wenyan S, Zixuan N, Pai Z (2021). Design concept evaluation of smart product-service systems considering sustainability: An integrated method. Comput. Ind. Eng..

[42] Qi J, Hu J, Huang HQ, Peng YH (2022). New customer-oriented design concept evaluation by using improved Z-number-based multi-criteria decision-making method. Adv. Eng. Inform..

[43] Zhou TT, Chen ZH, Ming XG (2022). Multi-criteria evaluation of smart product-service design concept under hesitant fuzzy linguistic environment: A novel cloud envelopment analysis approach. Eng. Appl. Artif. Intell..

[44] Huang GQ, Xiao LM, Zhang GB (2023). An integrated design concept evaluation method based on best-worst entropy and generalized TODIM considering multiple factors of uncertainty. Appl. Soft Comput..

[45] Yang Q (2024). Concept design evaluation of sustainable product-service systems: A QFD-TOPSIS integrated framework with basic uncertain linguistic information. Group Decis. Negot..

[46] Barfod MB, van den Honert R, Salling KB (2016). Modeling group perceptions using stochastic simulation: Scaling issues in the multiplicative AHP. Int. J. Inf. Technol. Decis. Making.

[47] Chen Z, Zhong P, Liu M, Ma Q, Si G (2022). A novel integrated MADM method for design concept evaluation. Sci. Rep..

[48] Ma Q, Sun H, Chen Z, Tan Y (2023). A novel MCDM approach for design concept evaluation based on interval-valued picture fuzzy sets. PLoS ONE.

[49] Fan JP, Zhang H, Wu MQ (2022). Dynamic multi-attribute decision-making based on interval-valued picture fuzzy geometric heronian mean operators. IEEE Access.

[50] Cuong, B. C., Kreinovitch, V. & Ngan, R. T. 19–24.

[51] Zulkifli N, Abdullah L, Garg H (2021). An integrated interval-valued intuitionistic fuzzy vague set and their linguistic variables. Int. J. Fuzzy Syst..

[52] Chen Z, Zhong P, Liu M, Sun H, Shang K (2021). A novel hybrid approach for product concept evaluation based on rough numbers, shannon entropy and TOPSIS-PSI. J. Intell. Fuzzy Syst..

[53] Göçer F (2021). A novel interval value extension of picture fuzzy sets into group decision making: An approach to support supply chain sustainability in catastrophic disruptions. IEEE Access.

