# Supplementary material for: An integrated design concept evaluation model based on interval valued picture fuzzy set and improved GRP method
Source: Sci Rep. 2024 Apr 10;14:8433. doi: 10.1038/s41598-024-57960-9 (PMC11006953; doi:10.1038/s41598-024-57960-9)
Supplement: Supplementary file 1 — Supplementary Information. [file 41598_2024_57960_MOESM1_ESM.docx]

**Appendix A:** DMs' judgements for alternatives with respect to criteria.

Decisions made by DM$R^{1}$

|  | A_1_ | A_2_ | A_3_ |
| --- | --- | --- | --- |
| $C_{1}$ | H | VH | H |
| $C_{2}$ | L | VH | VH |
| $C_{3}$ | H | H | VH |
| $C_{4}$ | H | H | M |
| $C_{5}$ | VH | M | H |
| $C_{6}$ | L | H | VH |
| $C_{7}$ | H | M | L |
| $C_{8}$ | L | L | M |

Decisions made by DM$R^{2}$

|  | A_1_ | A_2_ | A_3_ |
| --- | --- | --- | --- |
| $C_{1}$ | H | H | H |
| $C_{2}$ | VH | H | H |
| $C_{3}$ | H | H | VH |
| $C_{4}$ | VH | M | M |
| $C_{5}$ | H | M | H |
| $C_{6}$ | M | VH | H |
| $C_{7}$ | L | VH | M |
| $C_{8}$ | M | M | H |

Decisions made by DM$R^{3}$

|  | A_1_ | A_2_ | A_3_ |
| --- | --- | --- | --- |
| $C_{1}$ | H | H | VH |
| $C_{2}$ | L | H | H |
| $C_{3}$ | H | M | H |
| $C_{4}$ | H | M | M |
| $C_{5}$ | H | L | H |
| $C_{6}$ | M | VH | H |
| $C_{7}$ | M | M | L |
| $C_{8}$ | L | H | H |

Decisions made by DM$R^{4}$

|  | A_1_ | A_2_ | A_3_ |
| --- | --- | --- | --- |
| $C_{1}$ | H | M | H |
| $C_{2}$ | H | L | VH |
| $C_{3}$ | M | VH | VH |
| $C_{4}$ | VH | H | M |
| $C_{5}$ | H | H | M |
| $C_{6}$ | L | VH | H |
| $C_{7}$ | M | L | L |
| $C_{8}$ | L | H | H |

Decisions made by DM$R^{5}$

|  | A_1_ | A_2_ | A_3_ |
| --- | --- | --- | --- |
| $C_{1}$ | H | H | M |
| $C_{2}$ | H | H | M |
| $C_{3}$ | M | VH | H |
| $C_{4}$ | H | M | H |
| $C_{5}$ | M | H | H |
| $C_{6}$ | L | H | VH |
| $C_{7}$ | H | M | L |
| $C_{8}$ | M | H | H |

Decisions made by DM$R^{6}$

|  | A_1_ | A_2_ | A_3_ |
| --- | --- | --- | --- |
| $C_{1}$ | H | H | H |
| $C_{2}$ | H | VH | M |
| $C_{3}$ | VH | L | M |
| $C_{4}$ | H | M | L |
| $C_{5}$ | H | H | H |
| $C_{6}$ | M | H | M |
| $C_{7}$ | M | M | L |
| $C_{8}$ | L | M | M |

Decisions made by DM$R^{7}$

|  | A_1_ | A_2_ | A_3_ |
| --- | --- | --- | --- |
| $C_{1}$ | H | H | H |
| $C_{2}$ | M | L | H |
| $C_{3}$ | M | L | VH |
| $C_{4}$ | H | M | L |
| $C_{5}$ | H | H | M |
| $C_{6}$ | M | H | H |
| $C_{7}$ | L | M | M |
| $C_{8}$ | M | L | H |

Decisions made by DM$R^{8}$

|  | A_1_ | A_2_ | A_3_ |
| --- | --- | --- | --- |
| $C_{1}$ | M | VH | H |
| $C_{2}$ | H | M | H |
| $C_{3}$ | H | H | M |
| $C_{4}$ | M | H | H |
| $C_{5}$ | H | H | H |
| $C_{6}$ | H | VH | H |
| $C_{7}$ | M | M | M |
| $C_{8}$ | L | M | H |

Decisions made by DM$R^{9}$

|  | A_1_ | A_2_ | A_3_ |
| --- | --- | --- | --- |
| $C_{1}$ | H | H | H |
| $C_{2}$ | VH | H | H |
| $C_{3}$ | H | M | M |
| $C_{4}$ | M | H | H |
| $C_{5}$ | M | H | H |
| $C_{6}$ | H | M | H |
| $C_{7}$ | H | H | M |
| $C_{8}$ | VH | M | H |

Decisions made by DM$R^{10}$

|  | A_1_ | A_2_ | A_3_ |
| --- | --- | --- | --- |
| $C_{1}$ | H | H | H |
| $C_{2}$ | H | H | VH |
| $C_{3}$ | M | VH | L |
| $C_{4}$ | M | L | M |
| $C_{5}$ | H | M | H |
| $C_{6}$ | M | H | H |
| $C_{7}$ | L | H | VH |
| $C_{8}$ | H | VH | H |
